# Mammalian genomic regulatory regions predicted by utilizing human genomics, transcriptomics, and epigenetics data

**DOI:** 10.1093/gigascience/gix136

**Published:** 2018-02-16

**Authors:** Quan H Nguyen, Ross L Tellam, Marina Naval-Sanchez, Laercio R Porto-Neto, William Barendse, Antonio Reverter, Benjamin Hayes, James Kijas, Brian P Dalrymple

**Affiliations:** 1 CSIRO Agriculture, 306 Carmody Road, St. Lucia, 4067, QLD, Australia; 2 Divisions of Genomics of Development and Disease, Institute for Molecular Bioscience, University of Queensland, 306 Carmody Road, St. Lucia, 4067, QLD, Australia; 3 School of Veterinary Science, University of Queensland, Veterinary Science Building (8114), Gatton, 4343, QLD, Australia; 4 The Queensland Alliance for Agriculture and Food Innovation (QAAFI), University of Queensland, 306 Carmody Road, St Lucia, 4067, QLD, Australia; 5 Institute of Agriculture, The University of Western Australia, 35 Stirling Highway, Crawley, Perth, Western Australia, 6009, Australia

**Keywords:** regulatory genomics, mammalian genome, cattle, pigs, enhancers, promoters, transcription factors, SNP, *PLAG1, Poll*

## Abstract

Genome sequences for hundreds of mammalian species are available, but an understanding of their genomic regulatory regions, which control gene expression, is only beginning. A comprehensive prediction of potential active regulatory regions is necessary to functionally study the roles of the majority of genomic variants in evolution, domestication, and animal production. We developed a computational method to predict regulatory DNA sequences (promoters, enhancers, and transcription factor binding sites) in production animals (cows and pigs) and extended its broad applicability to other mammals. The method utilizes human regulatory features identified from thousands of tissues, cell lines, and experimental assays to find homologous regions that are conserved in sequences and genome organization and are enriched for regulatory elements in the genome sequences of other mammalian species. Importantly, we developed a filtering strategy, including a machine learning classification method, to utilize a very small number of species-specific experimental datasets available to select for the likely active regulatory regions. The method finds the optimal combination of sensitivity and accuracy to unbiasedly predict regulatory regions in mammalian species. Furthermore, we demonstrated the utility of the predicted regulatory datasets in cattle for prioritizing variants associated with multiple production and climate change adaptation traits and identifying potential genome editing targets.

## Background

Predicting functional features of the genome beyond protein-coding regions has been the primary focus of the post-genome sequencing era [1, 2]. More than 90% of common genetic variants associated with phenotypic variation of complex traits are located in intergenic and intronic regions that regulate gene expression but do not change protein structure [3–5]. Moreover, SNPs associated with diseases such as autoimmune diseases, multiple sclerosis, Crohn's disease, rheumatoid arthritis, and type 1 diabetes are strikingly enriched in promoters and enhancers [4, 6, 7]. Annotation of functional regions of the genome that harbour SNPs identified by genome-wide association studies (GWAS) to be significantly associated with variation in phenotype will contribute to the identification of functional SNPs and causative mutations, thereby suggesting genetic targets and markers for numerous applications in human health care and agricultural livestock production [8–11].

However, in mammalian species other than the human and mouse, there are few data available at the genome level for discovery of regulatory elements. The recently established Functional Annotation of ANimal Genomes (FAANG) consortium has begun to address this deficiency in a coordinated fashion [12, 13]. It is expected that core assays identifying regulatory elements for key tissues in a number of production animals will be produced by the FAANG consortium and collaborators. However, the information generated in the foreseeable future for livestock is likely to remain far less comprehensive for coverage of tissues, sampling conditions, and breadth of annotation of regulatory elements compared with the human and mouse. The deficiency in the genome-wide prediction of regulatory elements is far greater for most other mammalian species. We have developed a computational method to utilize thousands of human regulatory datasets to predict regulatory elements in important mammalian species.

Transcriptional regulatory DNA elements (RDEs) are defined as genomic regions that are binding sites for 1, or usually a combination of, transcription factors (TFs) and transcriptional coregulators [14–16]. Across distant species from *C. elegans* to *D. melanogaster* to humans, the architecture of gene regulatory networks, organization of chromatin topological domains, chromatin context at enhancer and promoter regions, and nucleosome positioning are remarkably conserved [17, 18]. For example, the majority of co-associations of transcription factors (i.e., combinations of different transcription factors binding to the same genomic region) at proximal transcription start site regions in humans remain proximal in the worm (80%) and fly (100%). Large-scale comparisons between the human and mouse (*M. musculus)* in the Encyclopedia of DNA Elements (ENCODE) project found a high level of conservation of binding motifs and activities, including TF binding to different chromatin states (*r* = 0.9), proportion of enhancers in TF binding regions (*r* = 0.7), DNA methylation preferences within TF-occupied regions (hypomethylated regions in both species), and TFs sharing a conserved primary binding motif sequence (∼94% of studied TFs) [19]. The human Encyclopedia of DNA Elements (ENCODE), Functional Annotation of the Mammalian genome (FANTOM), Roadmap Epigenomics Mapping Consortium (ROADMAP), and related projects have generated large volumes of data relevant to the identification of promoters, enhancers, and other RDEs [6, 20, 21]. However, these data have not been utilized for predicting regulatory genomic regions in other mammalian species—a strategy that can produce more comprehensive predictions than alternative options using a small set of experimental assays to identify a part of the regulatory repertory in the targeted species. We recognize that species-specific regulatory elements may be underrepresented in this process. However, we note that the fundamental biology of, e.g., that encompassing developmental programs, response to stimuli, reproduction, energy homeostasis, and many other systems shows considerable conservation of components and processes across species [17, 19, 22].

In the current research, we developed the Human Projection of Regulatory Regions (HPRS) method to utilize results from thousands of biochemical assays in human samples to computationally predict equivalent information in other mammalian species. The method exploits the conservation of regulatory elements at the DNA sequence and genome organizational levels to map these elements to other mammalian species. It then uses species-specific data to filter these mapped sequences, which are enriched for regulatory sequence features, to predict a set of high-confidence regulatory regions. We selected cattle as the target species to build the HPRS pipeline and then used the pig as a test species to validate the pipeline. The 2 species are important agricultural ruminant and nonruminant species, respectively, with genomes sequenced but with little information available about genomic regulatory regions [12]. We also applied the method to the genomes of 8 additional mammals. We demonstrated that the predicted regulatory dataset produced by the HPRS pipeline is useful for selecting more likely functional SNPs before (e.g., for SNP chip design) and after (e.g., for prioritizing significant SNPs) GWAS analysis, genomic prediction models, and the understanding of biological mechanisms underlying noncoding genomic variant effects to potentially identify regulatory targets for genome editing.

## Results and Discussion

### A pipeline for the projection of human genomic features to other mammals

**Figure 1:**
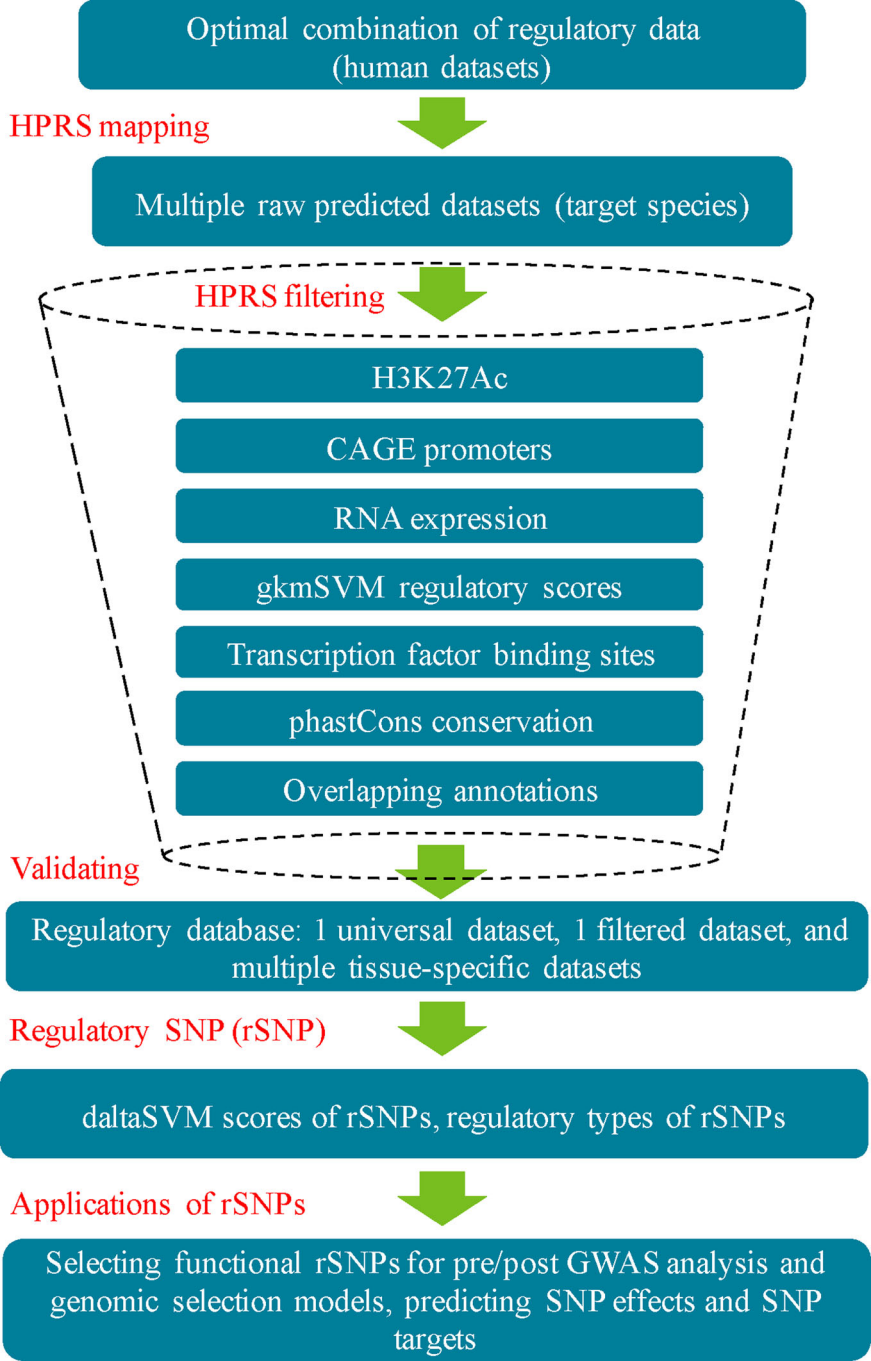
A streamlined workflow for the prediction of regulatory regions. Four key steps include mapping human regulatory regions to a target genome (creating a universal dataset), filtering the mapped regions by 7 epigenomic, transcriptomic, and genomic criteria to keep only regions with potential regulatory functions, validating the predicted regions by comparing with the known reference dataset, and translating the findings to potential applications in genomic technology.

The 4 key elements of the HPRS pipeline (Fig. 1) include (1) selection of suitable regulatory datatypes (biochemical assays) and tissues in humans; (2) mapping the selected features to the target species by utilizing conservation of genome organization and sequence identity to maximize coverage without compromising specificity; (3) first round filtering of the mapped regions to retain high-confidence mapped features, which had strict 1-to-1 forward and reciprocal mapping and where human features have multiple mappings to the target genome, keeping only those with high sequence identity; and (4) second round filtering by applying a pipeline to utilize available (often limited in scale and coverage) species-specific data to prioritize regions likely to be functional in the target species.

### Optimizing parameters for mapping sequence features across genomes

**Figure 2:**
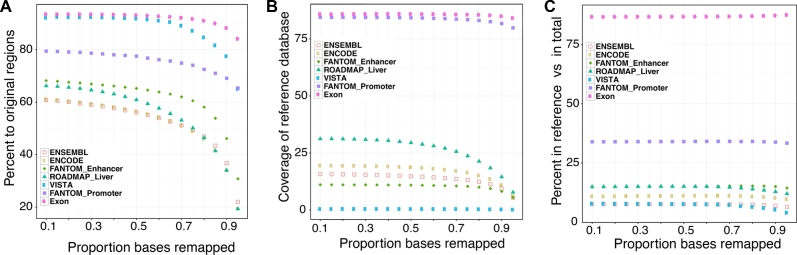
Optimization of mapping parameters using 7 input databases. The input databases included 5 human enhancer databases (ENSEMBL, ENCODE, ROADMAP liver tissue, Vista, and FANTOM enhancers), 1 human promoter database (FANTOM promoters), and 1 annotated human exon database (UCSC hg19) [6, 18, 21, 56, 57]. The numbers of regions from each dataset used to optimize parameters are shown in Table S4. We used the UCSC pair-wise whole-genome alignment chain files between the human genome (hg19) and the bovine genome (UMD3.1) and performed mapping from the human genome to the bovine genome (minMatch 0.1 to 0.95 as shown in the x-axis) and then reciprocal mapping from the bovine genome back to the human genome [52, 58–60]. A, Recovered rate, defined as the percentage of the number of mapped regions with exact reciprocal mapping to the total number of original regions in humans. B, Confirmation rate, defined as the percentage of reference regions covered by predicted regions to the total number in reference regions (Villar reference enhancers, Villar reference promoters, and cattle GENCODE genes V19). C, Specificity, defined as the percentage of matched reference (true positive for the reference dataset) compared with the total number of predicted regions.

To identify regions that were likely to be orthologous between genomes, we deployed the liftOver tool [23] and the precomputed alignment files available from UCSC to map regulatory regions in the human genome to the cattle genome based on sequence similarity and genome location. First, we optimized the minMatch mapping threshold of the liftOver tool, which is the minimum proportion of bases to the total length of a region mappable to contiguous aligned segments in the target genome. The minMatch parameter was thoroughly tested with a range from high stringency 0.95 down to 0.1 (Fig. 2). The minMatch parameter values were assessed using 7 diverse datasets (Fig. 2, Table 1).

**Table 1:** Summary information for the optimized set of human regulatory datasets used for HPRS mapping^a^

Dataset	Tissues/cell lines	Total No. of regions	Region types	Mean length, bp
ENCODE Distal TFs [19, 26]	0/91	1 122 364	Binding sites for 163 TFs	151.2
ENCODE Proximal TFs [19, 26]	0/91	384 343	Binding sites for 163 TFs	151.4
ROADMAP^b^ [21]	24 primary cells (e.g., blood cells, immune cells, and breast myoepithelial cells), 14 primary culture (e.g., skin, muscle satellite, neurosphere, bone marrow), and 50 primary tissues (e.g., thymus, spleen, lung, fetal stomach)	9 102 278	Enhancers	970.8
FANTOM Enhancers [6, 25]	135/673	43 011	Enhancers	289
FANTOM Promoters [20, 25]	152/823	201 802	Promoters	21.5

^a^Information on data types and models is described in Table S3.

^b^See Table S5 for sample source details.

The percentage of regions mappable to the target genome was compared with the total number of elements in the human regulatory databases (Fig. 2A). For cattle, mappable regions were defined as: (1) a small sequence segment (SSS) that can be mapped from the human to the bovine genome; (2) the resulting SSS can be mapped back (reciprocally mapped) from the bovine to the human genome; and (3) the boundaries of the reciprocally mapped SSS were within 25 bp of the boundaries of the original SSS in the human genome.

In all 5 enhancer datasets tested as shown in Fig. 2A, the ratio of mapped regions increased steadily when the minMatch parameter was reduced from 0.95 to 0.55, with a much slower increase when the minMatch was reduced from 0.55 to 0.10 (Fig. 2A). The accuracy of the sequence projection was assessed as the percentage of mapped regions that overlapped with a feature present in a reference cattle liver enhancer dataset, identified experimentally by histone 3 lysine 27 acetylation (H3K27Ac—a marker for active enhancers) and histone 3 lysine 4 trimethylation (H3K4me3—a marker for active promoters near transcription start sites) assays (hereafter referred to as the Villar reference datasets) (Fig. 2B) [22]. The coverage of the relevant reference datasets (Villar reference promoters, Villar reference enhancers, and UCSC exons) also increased when the minMatch was reduced for some, but not all databases (Fig. 2B). Importantly, the reduction in mapping threshold did not lead to a loss of specificity, which is defined as the percentage of predicted enhancers that matched Villar reference enhancers (true positive for the reference dataset) compared with the total number of enhancers predicted using the particular input dataset (Fig. 2C). The combined results shown in Fig. 2A and Fig. 2B suggest that reducing minMatch to lower than 0.55 still increases (at a slower rate) the number of mapped regions (for the ROADMAP, ENSEMBL, FANTOM, and ENCODE datasets) (Fig. 2B) and increases the chance of detecting more reference enhancers (for the ROADMAP, ENSEMBL, and ENCODE datasets (Fig. 2A). No significant difference was observed when lowering the minMatch from 0.2 to 0.1, but a slight gain in the percentage of mappable regions was obtained when decreasing the minMatch from 0.3 to 0.2. Therefore, the parameter testing indicated that the optimal minMatch threshold was 0.2.

We also developed the method to detect regions possibly from gene duplication events (Supplementary Methods). To identify regions possibly resulting from duplication events (Fig. S1A), the HPRS mapping pipeline pooled unmapped regions in the human datasets (with minMatch = 0.2) and mapped regions with no exact reciprocal matches for a second round mapping with different parameters (allowing multiple mappings and keeping only results with similarity higher than 80%) to rescue regions with multiple map targets.

### Optimized use of human regulatory datasets

**Figure 3:**
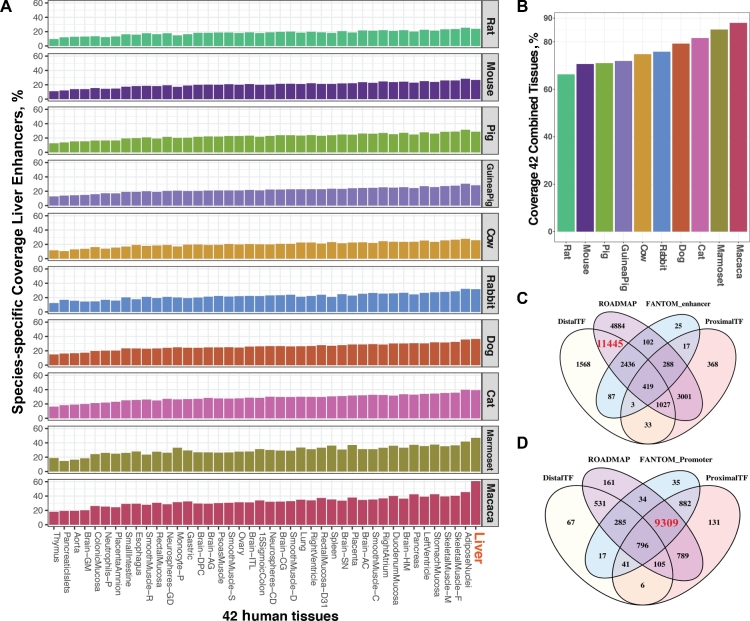
Effects of combining databases. A, Application of the HPRS mapping method to the genomes of 10 mammalian species. The input for the HPRS mapping was 1 of the 42 ROADMAP human datasets (38 adult tissues and 4 cell lines as shown in the x-axis) [22] to 10 mammalian species. The Villar reference enhancer datasets determined by H3K27Ac and H3K4me3 assays for liver tissue in each species were used to estimate the coverage of experimental enhancers by the predicted dataset (shown in the y-axis as species-specific enhancer dataset). For each species, the coverage was the percentage of the Villar reference enhancer dataset that overlapped with the HRPS prefiltered enhancers. B, The combination of all 40 tissues in each species was used. C and D, The optimal combination of 5 databases for enhancers and promoters, respectively. The reference datasets include ROADMAP enhancers (42 tissues), ENCODE distal TFs, ENCODE proximal TFs, FANTOM enhancers, and FANTOM promoters. The numbers shown in the intersections are the number of common regulatory regions between the HPRS mapped regions and the Villar reference datasets.

Regulatory regions can be active or quiescent, depending on the cell type and the biological state, and therefore prediction using a single tissue/cell line, or a single assay type, is unlikely to produce a high coverage of all possible regulatory sequences of a species [24]. Therefore, we investigated the effect of using different databases on the predictive capacity of HPRS. First, we compared the mapping coverage of enhancers from 42 human ROADMAP datasets [21] with the reference liver enhancer datasets, which were experimentally identified (by H3K27Ac assay for liver tissues) for 10 mammalian species reported in Villar et al. (Fig. 3A and B, Tables 1 and 5) [22]. Fig. 3A shows the percentage of Villar reference enhancers (e.g., enhancers detected in liver tissues in cats) that overlap with HPRS-predicted regions by mapping each of the original 42 human tissues to the targeted species (e.g., to the cat genome). Fig. 3B shows the percentage overlapping with the results from using the combined 42 tissues. Comparing results from each tissue, or from combined tissues in each species, enabled assessment of variation due to evolutionary distance or tissue specificity. Second, we evaluated the predictions from human to bovine based on different datatypes, including promoter databases (FANTOM), enhancer databases (FANTOM and ROADMAP), and transcription factor binding site databases (ENCODE proximal and distal TFs) (Fig. 3C and D). Each of the datatypes has unique sequence features that define different types of regulatory regions, e.g., those that are specific to promoters or enhancers. In general, species with closer evolutionary distance to humans had more HPRS-predicted enhancers matching the relevant Villar reference liver datasets (Fig. 3A). For each tissue, the relative mapping rates were similar between species. Between different tissues across the 42 ROADMAP datasets, thymus enhancers had the lowest mapping rate and liver enhancers the highest mapping rate in most species (Fig. 3B). Notably, the tissue specificity effect, exemplified by the higher mapping rate for ROADMAP liver datasets to the relevant species Villar reference datasets than for other ROADMAP tissues (Fig. 3B), was reduced substantially if the 2 primates that were more evolutionarily related to humans (macaque and marmoset) were removed from the comparison.

As the coverage of the reference cattle liver enhancer dataset was not significantly higher with human liver enhancers than with enhancers from many of the other human ROADMAP tissue enhancer datasets [21], we asked whether combining tissues would increase coverage. By combining the predictions from the 42 ROADMAP datasets, 2- to 4-fold higher coverage than from 1 tissue alone (at least 60% total coverage) could be obtained across a variety of species, with coverage being lowest for the rat and highest for macaque (Fig. 3A and B). Furthermore, we found that separate databases constructed using different models and biochemical assays were complementary, and combining them significantly increased coverage compared with a single database alone (Fig. 3C and D). For example, prediction using the ENCODE distal TF dataset and the ROADMAP enhancer dataset covered the highest number of Villar cattle reference enhancers, while prediction using the FANTOM promoter and the ENCODE proximal TFBS databases covered more Villar cattle reference promoters, and each dataset could add a number of unique regulatory regions not found in other datasets (Fig. 3C and D). The combination of 88 ROADMAP datasets [21], the FANTOM enhancer and promoter datasets [25], and the ENCODE distal and proximal TF datasets [26] generated a maximum enhancer coverage of 95% (for macaque) and promoter coverage of 98% (for marmoset). Therefore, we selected an optimal combination of human input databases for the HPRS pipeline on the basis that they represent promoters, enhancers, and TFBS from a large combination of human tissues and primary cells and were generated by different methods (Table 1).

### Predicting promoters

One of the most comprehensive human promoter datasets is the FANTOM5 promoter atlas generated experimentally by CAGE data from almost 1000 tissues and cell lines [20]. CAGE is a sensitive methodology for the detection of transcription start sites (TSS) and hence defines core promoter regions where there is binding of the transcriptional machinery [27]. Promoters generally have a high concentration of TFBS, typically within 300 bp upstream and 100 bp downstream of the TSS [20]. Promoter sequences are more evolutionarily conserved than enhancer sequences, and therefore a larger proportion can be mapped from human to other mammal genomes [22].

Of 201 802 CAGE transcription initiation peaks in the FANTOM5 human promoter atlas [20], 154 377 (76.5% of the total) were mappable to the bovine genome (Table 2). The HPRS using CAGE predicted new TSS not present within the existing bovine genome annotation (Ensembl Build 85). Although a promoter dataset for cattle can be inferred by defining upstream sequences of genes with annotated TSS, this indirect inference results in a small number of promoters. Approximately 26 740 cattle genes (coding, lncRNAs, miRNAs, etc.) in the reference dataset used (Ensembl Build 85) have annotated TSS. This dataset is far from comprehensive because of the expected underrepresentation of noncoding genes and of alternative promoters (APs). The 1 gene-one promoter and 1 gene-one protein concepts are no longer appropriate to describe the diverse transcriptome [28]. APs are common and are functionally important. A number of APs were found associated with complex traits [29]. While 51% of the Ensembl cattle TSS are covered by mapped human CAGE transcription initiation peaks (3.7 Mb), only 38.4% are covered by the experimentally defined promoters (32.9 Mb) in Villar et al. [22], suggesting that HPRS predictions based on human CAGE data could enrich promoter coverage in the cow by more than 12 times compared with the standard promoter assay (H3K4me3 ChIP-Seq) (Table 2). Active TSS regions from 88 human tissues in the ROADMAP were mapped to 81 892 putative promoters in cattle [21], with a total length of 135.6 Mb. Noticeably, the average number of Ensembl reference TSS that overlapped every 1 Mb of predicted promoters based on the ROADMAP database was 37-fold lower than those based on the CAGE database (Table 2).

**Table 2:** Summary of promoter predictions

Dataset	Total regions in cattle (MB, %)^a^	Overlap with Villar^b^ dataset (%)	Fold enrichment of Villar dataset	No. within 200 bp of TSS (%)^c^	Fold enrichment of TSS
Total No. of CAGE regions	154 377 (3.68, 0.138)	11 606 (84.1)	609	13 676 (51.0)	370
Filtered set CAGE regions	145 912 (3.46, 0.129)	11 203 (81.2)	629	13 011 (48.7)	377
Total all regulatory regions (Universal Dataset)	542 756 (937.39, 35.11)	13 329 (96.6)	3	20 759 (77.6)	2
Filtered regulatory regions (Filtered Dataset)	245 384 (356.1, 13.33)	13 104 (95.0)	7	17 715 (66.2)	5
Villar reference promoters	13 796 (32.90, 1.23)	13 796 (100)	NA	10 212 (38.2)	31
ROADMAP promoters	81 892 (135.6, 5.08)	12 677 (91.9)	18	14 388 (53.8)	11

^a^The total number of regions with liftOver at minMatch 0.2 and exact reciprocal matches, combined with regions that had multiple matches (no 1-to-1 relationship) but had high conservation (80% identity). The original human regions are from the FANTOM promoter dataset. The percentage was calculated for total genome size.

^b^Villar promoter dataset for cattle [22].

^c^Promoter count within 200 bp of the Ensembl annotated UMD3.1 TSS Ensembl build 85 (total 26 740).

HPRS using the CAGE dataset can predict many TSS at single-nucleotide resolution and can accurately predict transcriptional orientation. TSS are presented in the Ensembl database as single nucleotide genomic positions. HPRS-predicted promoters based on CAGE had exact overlap to the 7191 Ensembl TSS for cattle. While promoter prediction by using histone marks (such as those used by ROADMAP) cannot directly define transcriptional orientation, this information, predicted by HPRS using human CAGE data, is highly accurate [20]. Consistently, we found that of 13 676 genes that have TSS within 500 bp of mapped CAGE peaks, 96.9% (13 257) of genes had the same transcriptional orientation in the Ensembl annotation and predicted by human CAGE data. We therefore assigned promoter orientation using the predictions from the CAGE dataset.

### Mapping enhancer datasets

Prediction of enhancers is likely to be more challenging than predicting promoters because (1) enhancers are less conserved in DNA sequence; (2) enhancer locations evolve faster [19, 22]; and (3) enhancer effects are usually independent of the distance, orientation, and relative location (upstream or downstream) of gene targets [14]. To predict a broad set of sequences in a species that are active in 1 or more tissues or conditions, we expanded the human enhancer datasets to include 88 tissues, primary cell lines, and primary cell cultures generated by the ROADMAP project (Table 1) [21]; all human active enhancers defined by CAGE data from hundreds of tissues and cell lines in the FANTOM project [6]; and all the Villar experimentally defined reference cattle liver enhancers (Table 1) [22]. Cumulatively, the HPRS pipeline mapped more than 9.1 million human enhancer sequences to more than 5.9 million regions in the bovine genome, which were then merged into 542 756 nonoverlapping regions (Table 3). The merged dataset (Universal Dataset) covered 86% (excluding merged regions resulting from the original Villar reference enhancers) of the Villar enhancer reference dataset (Table 3). The term “Universal” reflects the initial pooling of all relevant human regulatory datatypes and datasets to form a large collection of genomic regions to be mapped to the target species. Regulatory sequences are often active in certain conditions, and remain inactive in most other cases. Therefore, pooling active regulatory regions from a large number of datasets can likely cover most active and inactive regulatory sequences, thus enabling the prediction of a Universal Dataset.

**Table 3:** Summary of mapped and filtered regulatory sequences

	No. of mapped regions (%)			Genome coverage, Mb (%)		
Datasets	Human	Cow	Pig	Human	Cow	Pig
Total genome size, Mb	NA^a^	NA	NA	3137.2 (100)	2670.4 (100)	2808.5 (100)
ROADMAP enhancers, % mapped to target species	9 102 278 (100)	5 917 129 (65)	5 620 417 (62)	8836.6^b^ (NA)	6142.4^b^ (NA)	5809.5^b^ (NA)
ROADMAP enhancers (overlapping regions were merged)	494 583 (100)	371 295 (75)	361 682 (73)	1123.2 (35.8)	885.6 (33.2)	826.2 (29.4)
FANTOM CAGE enhancers	43 011 (100)	34 303 (80)	27 558 (64)	12.4 (0.40)	12.2 (4.6)	9.6 (0.34)
ENCODE distal TFs	1 122 364 (100)	749 572 (67)	716 515 (64)	169.7 (5.4)	132.0 (4.9)	124.4 (4.4)
FANTOM CAGE promoter peaks	201 802 (100)	154 377 (76)	153 893 (76)	4.3 (0.14)	3.7 (0.14)	3.7 (0.13)
ENCODE proximal TFs	384 343 (100)	298 554 (78)	279 774 (73)	58.2 (1.9)	48.0 (1.8)	48.9 (1.7)
Merged ROADMAP, ENCODE, and FANTOM datasets (Universal Dataset)^c^	760 702	542 756 (86.1 and 96.6)^d^	519 913 (89.2 and 97.1)^d^	1165.7 (37.2)	919.5 (34.4)	857.8 (30.5)
Filtered Dataset	NA	245 384 (73.5 and 95.0)^d^	151 523 (69.8 and 95.6)^d^	NA	356.1 (13.3)	311.5 (11.1)

^a^NA, not applicable. The percentage was not calculated for these 3 values because overlapping regions are present in the different enhancer datasets.

^b^The total size is bigger than the genome size because overlapping regions are included.

^c^Total size of nonoverlapping regions in the Universal Dataset (before filtering); the percent overlapping Villar reference enhancers (the former) and promoters (the later) in the targeted species.

^d^Percent overlap Villar reference liver enhancers and promoters in the filtered datasets.

The HPRS mapping of the enhancer datasets predicted a large set of homologous regions that are potentially regulatory regions in cattle (the Universal Dataset). We noted that the alignability of DNA sequence does not automatically imply functionality [22], and therefore we applied a filtering pipeline to incorporate other types of cattle-specific data to prioritize regions more likely to have transcription regulation functions. The filtering pipeline used a combination of sequence features and epigenetics marks to enrich for likely regulatory enhancers and promoters, as discussed in the filtering section.

### Mapping transcription factor binding site datasets

To include potential regulatory regions beyond typical promoter and enhancer classifications, we performed HPRS mapping of human experimentally defined ENCODE TFBS (ENCODE annotation version 2) to the bovine genome. The ENCODE TFBS database contains binding sites for 163 key TFs, some of which represent additional types of regulatory regions other than enhancers and promoters (Table 1) [30]. The use of these TFBS datasets not only supported predictions from using the enhancer and promoter datasets, but more importantly, added other regulatory categories into the combined prediction of regulatory regions. For example, the binding targets of the CCCTC-binding factor (CTCF) are likely insulator regions [31], while enhancer of zeste homolog 2 (EZH2) binding sites may mark polycomb repressor complex 2 (PRC2) regions [32]. These ENCODE TFBS were identified as binding regions of TFs to nucleosome-free regions (∼151 bp per region), which are more biologically relevant than *de novo* scanning of genome sequence for TFBS based on short position weight matrices (PWMs; typically 6–12 bp) because the later method only uses DNA sequence and does not take into account the biological chromatin context, which is essential for transcription factor binding [33, 34]. In total, from the ENCODE TFBS dataset [26, 34], 298 554 proximal TFBS (total 47.97 Mb) and 749 572 distal TFBS (total 132.04 Mb) were projected by HPRS onto the bovine genome. We also show that the HPRS prediction using ENCODE transcription factor datasets was supported by 2 other independent prediction approaches (Supplementary Methods).

### The filtering pipeline for a high-confidence regulatory region dataset

**Figure 4.**
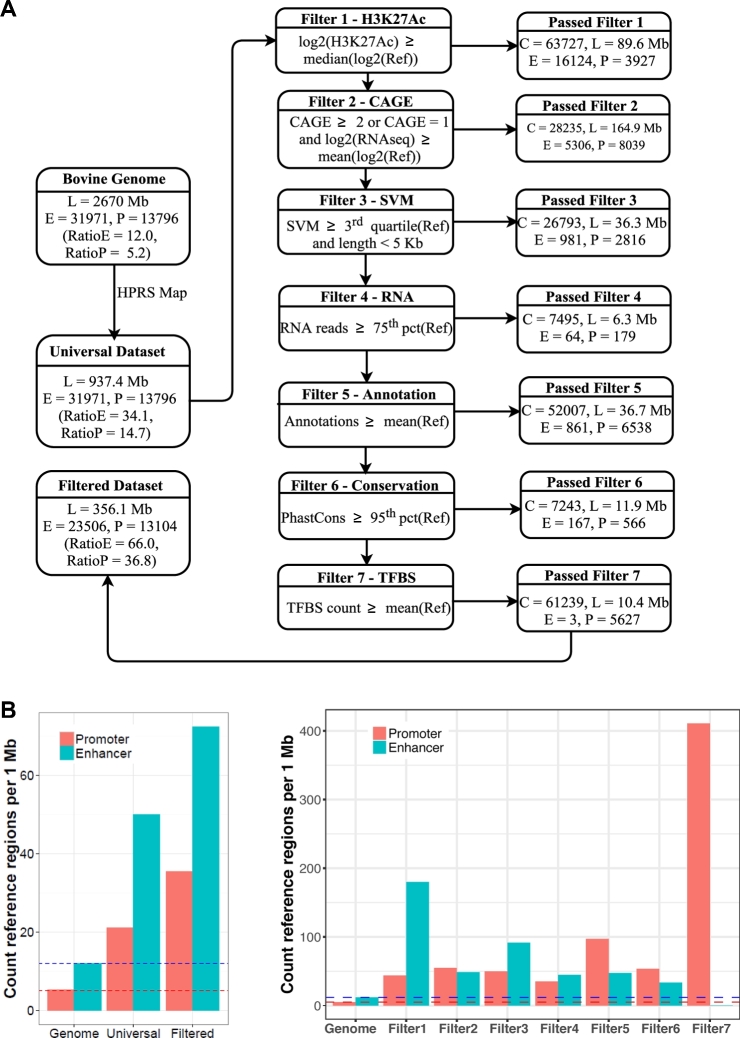
Enrichment of the enhancers and promoters by the filters in the HPRS filtering process. A, A pipeline to filter predicted regulatory regions from the Universal Dataset with 542 756 regions, covering 937.4 Mb of the genome (35.1%). The initial number of experimentally defined Villar reference datasets included 31 971 enhancers (E) and 12 257 promoters (P). The number of reference E and P, total number of predicted regulatory regions, and total length (in Mb) for all promoters and enhancers passing each filtering layer are shown. The Ratio_E_ (total enhancers overlapping Villar reference enhancers/total length) and Ratio_P_ (total promoters overlapping Villar reference promoters/total length) were used as criteria to assess enrichment for each filter. The 7 filters are described in Table 4 and in the Supplementary Methods. B, Two bar graphs showing enrichment results (using the same starting set) of using each of the 7 filtering steps in comparison with the baseline (whole genome) as shown in the dashed lines, and the Universal Dataset (mapped regions, not filtered). The y-axis shows the average number of reference promoters or enhancers in every 1 MB of the genome. The density of regulatory regions predicted is an indicator of the prediction coverage and accuracy. The higher values indicate more experimentally validated enhancers and promoters are enriched after filtering, suggestive of a more efficient filter. Each filter was tested independently, using the same Universal Dataset as the input, to compare the enrichment levels that resulted from each of the 7 filters.

The predictions produced by HPRS were optimized so that they occupied a relatively small part of the whole genome, but can universally predict regulatory regions in different cell types and tissues. Applying HPRS for selected datasets (Fig. 3, Table 1), we first produced a preliminary Universal Dataset and then refined it to generate a Filtered Dataset (Table 3). To remove redundancies, overlapping mapped ROADMAP enhancers (initially mapped separately for each of the 88 ROADMAP datasets) were merged (Table 3). Similarly, all mapped regions for promoters, merged enhancers, and TFBS with overlapping coordinates were merged into larger regions to form the final Universal Dataset (UD), containing 542 756 nonoverlapping regions. These regions covered 937.4 Mb (35.1%) of the bovine genome. The high coverage (35.1%) of the UD was due to the large collection of human datasets used as inputs for mapping to bovine (37.2% of the human genome) so that the UD covered almost all possible promoters, enhancers, and TFBS (Table 3). Importantly, the HPRS pipeline improves the specificity of the UD by applying a filtering step, which incorporates the power of cattle-specific data to predict a small set of potential transcription regulatory genomic regions in the bovine genome (Fig. 4, Table 4).

**Table 4:** Filters with species-specific data for selecting regulatory regions (refer to the Supplementary Materials and Methods)

		Length, Mb			Ratio of enhancers, count/Mb			No. of ratio promoters, count/Mb		
Filter	Filtering parameters	Cattle	Pig	Mouse	Cattle	Pig	Mouse	Cattle	Pig	Mouse
Whole genome	Genome baseline, enhancers/Mb	2670.4	2808.5	2730.8	31 971 (12.0)	23 804 (8.5)	18 396 (6.7)	13 796 (5.2)	11 114 (4.0)	15 164 (5.6)
Universal Dataset	Universal baseline, enhancers/Mb	937.4	882.4	699.7	31 971 (34.1)	23 804 (27.0)	18 396 (26.3)	13 796 (14.7)	11 114 (12.6)	15 164 (21.7)
CAGE	CAGE ≥ 2 or CAGE = 1 and RNAseq > mean(VillarRef)^a^	201.9	194.7	147.6	9628 (47.7)	6679 (34.3)	3935 (26.7)	10 152 (50.3)	9476 (48.7)	10 951 (74.2)
	CAGE ≥ 1	250.7	248	189.8	11 318 (45.2)	8214 (33.0)	5199 (27.0)	12 103 (48.3)	9936 (39.9)	12 722 (67.0)
H3K27Ac	Log2(H3K27Ac ≥ median(log2(VillarRef))^b^	89.6	103.8	47.0	16 124 (180.0)	11 985 (115.4)	6736 (143.3)	3927 (43.8)	9324 (89.8)	10 570 (225.0)
	Log2(H3K27Ac) ≥ mean(log2(VillarRef))	91.0	102.0	50.4	16 366 (179.8)	11 670 (114.4)	7255 (143.9)	3966 (43.6)	9305 (91.2)	10 707 (212.4)
RNAseq	Log2(RNAseq) ≥ 3rd quartile(log2(VillarRef))^c^	156.1	85.3	105.8	6999 (44.8)	3162 (37.1)	2726 (25.8)	5473 (35.1)	6412 (75.2)	4375 (41.3)
	Log2(RNAseq) ≥ median(log2(VillarRef))	278.1	184.0	193.1	12 147 (43.7)	6748 (36.6)	4626 (24.0)	8442 (30.4)	8709 (47.3)	6346 (32.9)
	Log2(RNAseq) ≥ mean(log2(VillarRef))	319.4	197.7	218.7	13 746 (43.0)	7268 (36.8)	5175 (23.7)	9249 (29.0)	8874 (44.9)	6839 (31.3)
gkm-SVM	Length < 3000 & SVM ≥ median(VillarRef)^d^	85.9	4.7	77.2	3603 (41.9)	261 (55.6)	3525 (45.7)	9208 (107.1)	359 (76.4)	8570 (110.0)
	Length < 3000 & SVM ≥ mean(VillarRef)	87.4	3.7	74.5	3645 (41.7)	200 (53.9)	3446 (46.3)	9230 (105.6)	287 (77.3)	8555 (114.9)
	Length < 5000 & SVM ≥ mean(VillarRef)	133.4	49.3	110.8	5673 (42.5)	1858 (37.6)	4285 (38.7)	9766 (73.2)	1333 (27.0)	9100 (82.1)
Annotation count	AnnCount ≥ 3rd quartile (VillarRef)^e^	72.8	41.4	362.4	3308 (45.5)	893 (21.6)	9183 (25.3)	9618 (132.2)	7489 (181.0)	11 317 (31.2)
	AnnCount ≥ median (VillarRef)	109.1	21.2	614.3	5173 (47.4)	887 (21.5)	13 366 (21.7)	10 599 (97.2)	7486 (181.7)	13 835 (22.5)
	AnnCount ≥ mean (VillarRef)	273.2	239.6	318.2	12 433 (45.5)	7792 (32.5)	8321 (26.2)	12 391 (45.4)	10 039 (41.9)	10 633 (33.4)
PhastCons	PhastCons ≥ 95th percentile (VillarRef)^f^	28.0	26.7	31.1	939 (33.5)	722 (27.1)	851 (27.3)	1504 (53.7)	1165 (43.7)	2177 (69.9)
	PhastCons ≥ median (VillarRef)	383.6	351.3	354.9	13 068 (34.1)	9425 (26.8)	8319 (24.4)	9929 (25.9)	7746 (22.1)	11 016 (31.0)
	PhastCons ≥ mean (VillarRef)	247.4	227.0	248.0	8415 (34.0)	6098 (26.8)	6039 (24.3)	8000 (32.3)	6249 (27.5)	9456 (38.1)
TFBS count	TFBScount ≥ median (VillarRef)^g^	16.3	12.9	213.3	4 (0.2)	2 (0.2)	6151 (28.8)	6700 (411.3)	3253 (252.0)	9739 (45.7)
	TFBScount ≥ mean (VillarRef)	379.9	882.4	295.9	12 933 (34.0)	23 804 (27.0)	7995 (27.0)	9956 (26.2)	10 788 (12.2)	10 729 (36.3)

^a^Regions with at least 2 CAGE peaks or with 1 CAGE peak, and the number of mapped reads from RNAseq data is higher than the mean mapped reads of the reference Villar enhancer dataset. The results are compared with results from applying another filtering criterion, which is CAGE peak above 1. The comparisons are based on total length of all selected regions, and the average count of the filtered regions normalized by length (regulatory regions per 1-Mb genome length).

^b^Regions with more of the H3K27Ac mapped reads than the median or mean mapped reads to reference enhancers in the Villar dataset (log2 scale).

^c^Regions with more of the RNAseq mapped reads than the 3rd quartile, median, or mean mapped reads to reference enhancers in the Villar dataset (log2 scale).

^d^Regions with length shorter than 3000 bp (or 5000 bp) and gkmSVM scores ≥ median or mean scores for the Villar enhancer dataset.

^e^Regions with more annotation terms (diversity of regulatory features) mapped to the regions, compared with those mapped to reference regions in the Villar enhancer dataset.

^f^Regions with higher conservation PhastCons scores compared with the 95th percentile, median, or mean scores to enhancers in the Villar dataset.

^g^Regions with more transcription factor binding sites than those in the Villar enhancer dataset.

The filtering pipeline reduced the UD to the much smaller Filtered Dataset (FD; the same as filtered UD), which covered a smaller part of the whole genome, but which still predicted most active enhancers and promoters (Table 4, Fig. 4). Detailed discussion on rationale for selecting each filter is in the Supplementary Materials and Methods. Briefly, the pipeline utilized both biological data in the target species (86 RNA-Seq datasets representing 79 cattle tissues [35], cattle H3K27Ac signal [22], and DNA sequence conservation scores) and computationally estimated criteria (gapped k-mers support vector machine (gkm-SVM) scores, number of overlapping annotations, and number of CB-predicted TFBS) (Fig. 4A).

Before filtering, the Universal Dataset had approximately 2.84 times higher Ratio_E_ (number of Villar reference enhancers by predicted regions divided by the length in Mb of predicted regions) and 2.82 times higher Ratio_P_ (similar to Ratio_E_, but for promoters) than the total genome baseline, and each filtering step in the pipeline increased Ratio_E_ and Ratio_P_ compared with the baseline (Fig. 4B, Table 4). At the end of the pipeline, a set of high-confidence regulatory regions (the FD), containing 245 384 sequences (with a total length of 356.1 Mb, equivalent to 13.3% of the whole genome) was obtained. The filtering reduced the number of regions by 2.2 times and the genome coverage by 2.6 times (Table 3, Fig. 4A), while still including most of the cattle liver reference enhancers and promoters (73.5% and 95.0%, respectively) (Table 4, Fig. 3A). Importantly, the filtered dataset had a 5.5 and 7.1 times higher Ratio_E_ and Ratio_P_, respectively, than the genome baseline (Fig. 4). The size and coverage of the bovine genome (356.1 Mb, 13.3%) by HPRS predicted regulatory regions were comparable to the published Fig. 1 for the mouse, which is 12.6% of the mouse genome, as predicted by ENCODE DNAse I accessibility data and transcription factor ChIP-Seq (using antibodies for 37 TFs on 33 tissues/cell lines) and histone modification ChIP-Seq data [2]. Similarly, applying the HPRS pipeline to the mouse genome, without using mouse-specific datasets from ENCODE or other sources (except for the reference Villar dataset), predicted potential regulatory regions that occupy 11.3% of the whole mouse genome.

### Validating and extending the HPRS pipeline in 9 other mammalian species

The performance of the HPRS pipeline was evaluated using the porcine (pig) genome (susScr3) [36]. HPRS had been developed based on the bovine genome, and the pig was then selected as a species for step-by-step comparison throughout the pipeline because of the availability of experimentally defined porcine promoter and enhancer reference datasets [22] and because the pig is an evolutionarily divergent nonruminant production animal. We obtained similar results in the pig compared with cattle on numbers of putative regulatory regions, percentage to total genome length, and coverage of the reference datasets (Tables 3 and 4). Importantly, we extended the application of the HPRS mapping data from the human to 8 additional mammalian species, which had reference promoter and enhancer datasets from the Villar et al. study. We generated HPRS mapped unfiltered UDs and observed consistently high coverage of the reference enhancer and promoter datasets, and the coverages were comparable between all 10 mammalian species (Table 5). Thus, the pipeline appears to have general utility, not just for livestock species, but also for mammals in general.

**Table 5:** HPRS predicted regulatory datasets for 10 species

Species	No. of regions	Total length, Mb	Enhancer coverage, %^a^	Promoter coverage, %^a^
Unfiltered datasets				
Cattle (bTau6)^b^	545 748	919.5	86.1	96.6
Pig (susScr3)	519 913	882.4	89.2	97.1
Marmoset (CalJac3)	642 144	1106.4	93.1	98.4
Rhesus Macaque (RheMac3)	693 312	1158.2	94.5	97.6
Dog (CanFam3)	570 317	877.5	89.4	97.6
Cat (FelCat5)	570 282	903.9	90.8	97.1
Guinea pig (CavPor3)	523 273	761.6	81.1	92.7
Rabbit (OryCun2)	531 109	819.4	86.8	96.8
Mouse (Mm10)	478 974	699.7	79.6	93.2
Rat (Rn5)	453 017	620.5	75.3	89.5
Filtered datasets^c^				
Cattle (bTau6)	245 358	356.1	73.5	95.0
Pig (susScr3)	151 523	311.5	69.8	95.6
Mouse (mm10)	281 071	308.4	68.9	91.4

The datasets were generated for each species using the same human data sources, including: 88 ROADMAP tissues/primary cell lines, FANTOM promoters and enhancers, and ENCODE proximal and distal TFs (Table S2) and combined with the Villar reference enhancer promoter dataset. The prediction results for each species are available as part of Additional file 2.

^a^Coverage of the relevant Villar reference datasets [22].

^b^Reference genomes are from UCSC [61].

^c^The relevant Villar reference species enhancer datasets were added prior to filtering.

### SNPs in regulatory regions are enriched for significant GWAS SNPs

**Figure 5:**
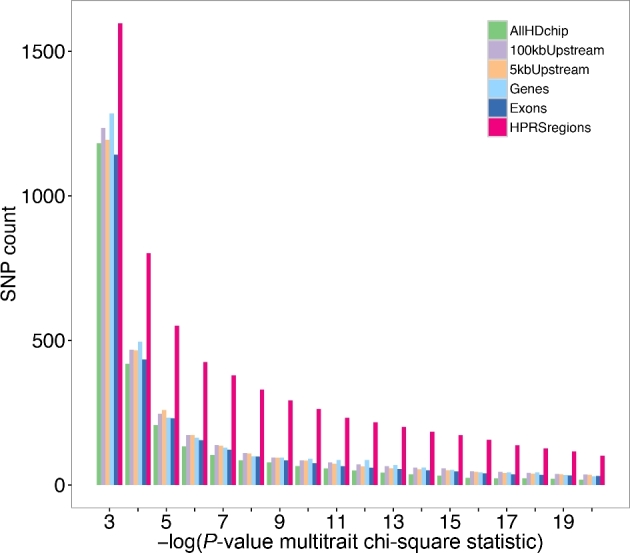
Enrichment of significant pleiotropic SNPs in regulatory genomic regions. Count of significant pleiotropic GWAS SNPs (*P*-values are from multi-trait meta-analysis chi-square test statistic for 32 traits) [37] in a set of ∼729 100 SNPs genotyped using the Illumina HD Bovine SNP chip or imputed from genotyping data from smaller Illumina Bovine SNP chips. Legend labels, from top to bottom: “AllHDchip”: 43 130 SNPs randomly selected (from all 692 529 SNPs on the HD chip, excluding those from chromosome X); “100kbUpstream”: 43 130 SNPs randomly selected (from 325 227 SNPs within 100 kb of upstream regions of coding genes); “5kbUpstream”: all 30 384 SNPs within the 5-kb upstream regions of coding genes (results scaled to 43k SNPs); “Genes”: 43 130 SNPs randomly selected (from 240 160 SNPs in coding genes); “Exons”: all 10 003 SNPs in exons of coding genes (results scaled to 43k SNPs); “HPRS regions”: 43 130 SNPs in regulatory regions.

More than 90% of significant GWAS SNPs lie outside gene-coding regions, and for those within the gene body (from the start to the termination site of the complete transcript, including introns), more than 92% are within intronic regions [3, 5]. To test the enrichment of potential causal SNPs within predicted regulatory regions in cattle, we explored the overlap between SNPs in regulatory regions and pleiotropic SNPs, which are SNPs significantly associated with multiple traits. The pleiotropic SNPs were identified by an independent GWAS study for 32 cattle feed intake, growth, body composition, and reproduction traits [37]. The GWAS used 10 191 beef cattle, with data (including imputed data) for 729 068 SNPs (Fig. 5). We observed a substantial fold enrichment (∼2–4 times) of SNPs with –log (*P-*value) from 3 to 20 in the Filtered Dataset compared with all other sets of commonly classifying SNPs in different genomic regions, including the set of SNPs 5 kb upstream of protein coding genes. We also observed higher counts (for 6 out of 10 traits) of associated SNPs within regulatory regions in a study on 10 climatic adaptation traits in 2112 Brahman beef cattle (Fig. S1) [38]. Similarly, we found enrichment of regulatory SNPs in a study of 5 major production and functional traits in 17 925 Holstein and Jersey dairy cattle (*P* < 0.05 for 3 out of 5 traits) (Table S1) [39]. These observations are consistent with the pipeline identifying regulatory SNPs from millions of SNPs in the genome and suggest that the predicted regulatory database is useful for prioritizing SNPs likely to be contributing to phenotypic variation of complex traits.

### The regulatory region datasets can be used to guide identification of potential causative SNPs and their gene targets

As examples of the application of our resources to identify likely causative mutations from a large list of significantly associated SNPs, we applied the HPRS approach to analyse 2 well-studied genetic variants in cattle that were known to contribute to phenotypic variation, but their mechanisms of action were not known because they were located within noncoding regions.

**Figure 6:**
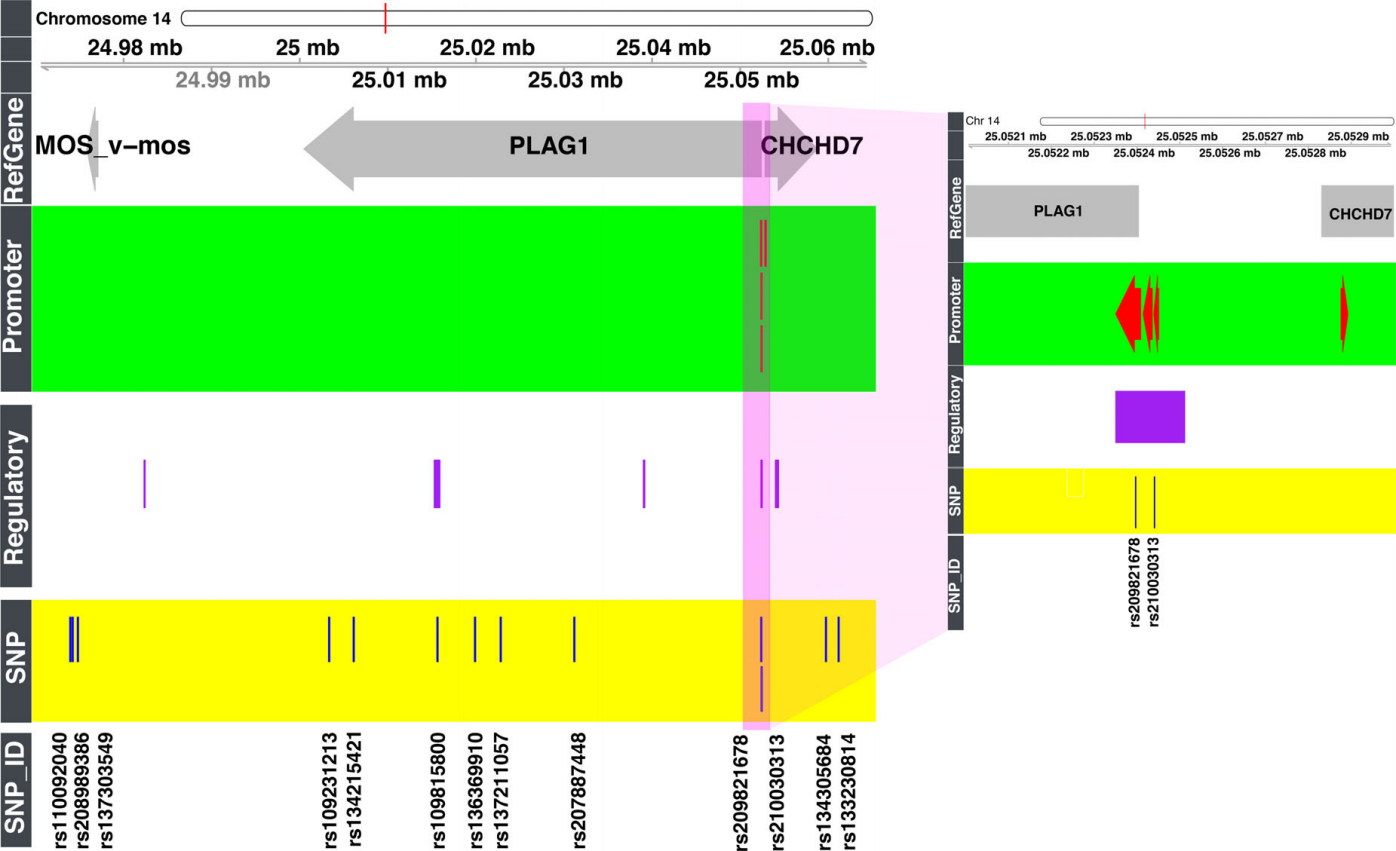
Application of the regulatory database to prioritize significant bovine SNPs identified by GWAS studies for functional validation. Overview of 13 significant SNPs fine-mapped by Karim et al. [40] is shown in the left panel. Among those SNPs, only 3 overlap regulatory regions and promoter regions in the predicted database. The right panel is a detailed view of the 2 SNPs validated as causative in Karim et al. [40]. Both SNPs are within promoter regions of the *PLAG1* gene, but not the *CHCHD7* gene. The regulatory (enhancers, promoters, and transcription factor binding sites) and promoter (only promoters) tracks display HPRS-predicted regions.

The bovine Pleomorphic adenoma gene 1 (*PLAG1*) locus has been identified in the control of stature (weight and height) by several independent GWAS studies in cattle [40, 41]. The study by Karim et al. [40] fine-mapped 14 SNPs associated with stature. The 14 SNPs are in the vicinity of *PLAG1* and the Coiled-coil-helix-coiled-coil-helix domain containing 7 (*CHCHD7*) gene, which are 540 bp apart (Fig. 6A). The 14 candidate SNPs are shown in Fig. 6A with coordinate locations relative to HPRS-predicted regulatory regions. The HPRS database suggests a strategy for further filtering these fine-mapped SNPs in 2 ways, first to prioritize gene targets and second to prioritize SNPs. The design of the validation experiment by Karim et al. [40] did not separate the 2 SNPs (rs209821678 and rs210030313) in the promoter region because both the long and short fragments used for activity assays in the study contained both SNPs. The HPRS prediction separates the 2 SNPs into 2 core CAGE peaks (Fig. 6B). The 2 peaks suggest 2 potentially separate binding sites of the transcriptional machinery. HPRS resolves the shared 540-bp promoter region into separate core promoter regions and suggests a new validation design in which 3 short, directional fragments focusing more specifically on core CAGE regions (2 near *PLAG1* and 1 near *CHCHD7*) can be used for functional assays of SNP genotype. Measuring promoter activity of these 3 constructs by using the similar promoter luciferase assay and transcription factor binding assay employed by Karim et al. [40] may confirm which of the 2 SNPs is causative and which gene is affected.

Furthermore, by applying a scoring model for regulatory variants, we generated deltaSVM score for each of 97 million known bovine SNPs (Supplementary Materials and Methods). The SNP rs209821678 had a deltaSVM score of –5.99. The score was beyond the 95th percentile range of SVM scores for 97 million SNPs, suggesting that it may play an important regulatory role. Notably, the rs209821678 deletion of the (CCG)x11 to (CCG)x9 trinucleotide repeats lies in a predicted G-quadruplex and may cause changes in its structure, an event that could alter transcriptional activity [42]. In contrast, the SNPs rs210030313 and rs109815800 did not have significant deltaSVM scores (0.51 and 3.2, respectively).

We then asked if the regions containing the SNPs interact with additional genes distant from the *PLAG1* locus. We applied HPRS for mapping interactions defined by chromatin conformation capture data (5C and Hi-C in the ENCODE human datasets) to predict distal targets of the promoter regions in the PLAG1 locus [43, 44], and we found that rs209821678 and rs210030313 are within the anchor A_447 043 (chr14: 25 044 319–25 054 287, UMD3.1), with a predicted target region (chr14: 25 478 861–25 497 096) near the Inositol monophosphatase domain containing 1 (*IMPAD1*). Variants within IMPAD1 have been implicated in short stature and chondrodysplasia (Table S2). Interestingly, the leading SNP identified in an analysis of pleiotropic genes affecting carcase traits in Nellore cattle, rs136543212 at chr14: 25 502 915, is slightly closer to *IMPAD1* [45]. The rs109815800 SNP, on the other hand, does not lie in any mapped Hi-C region. Together, the HPRS-predicted results strongly suggest that the rs209821678 variant is the causative SNP among the 14 candidates fine-mapped by Karim et al. [40].

**Figure 7:**
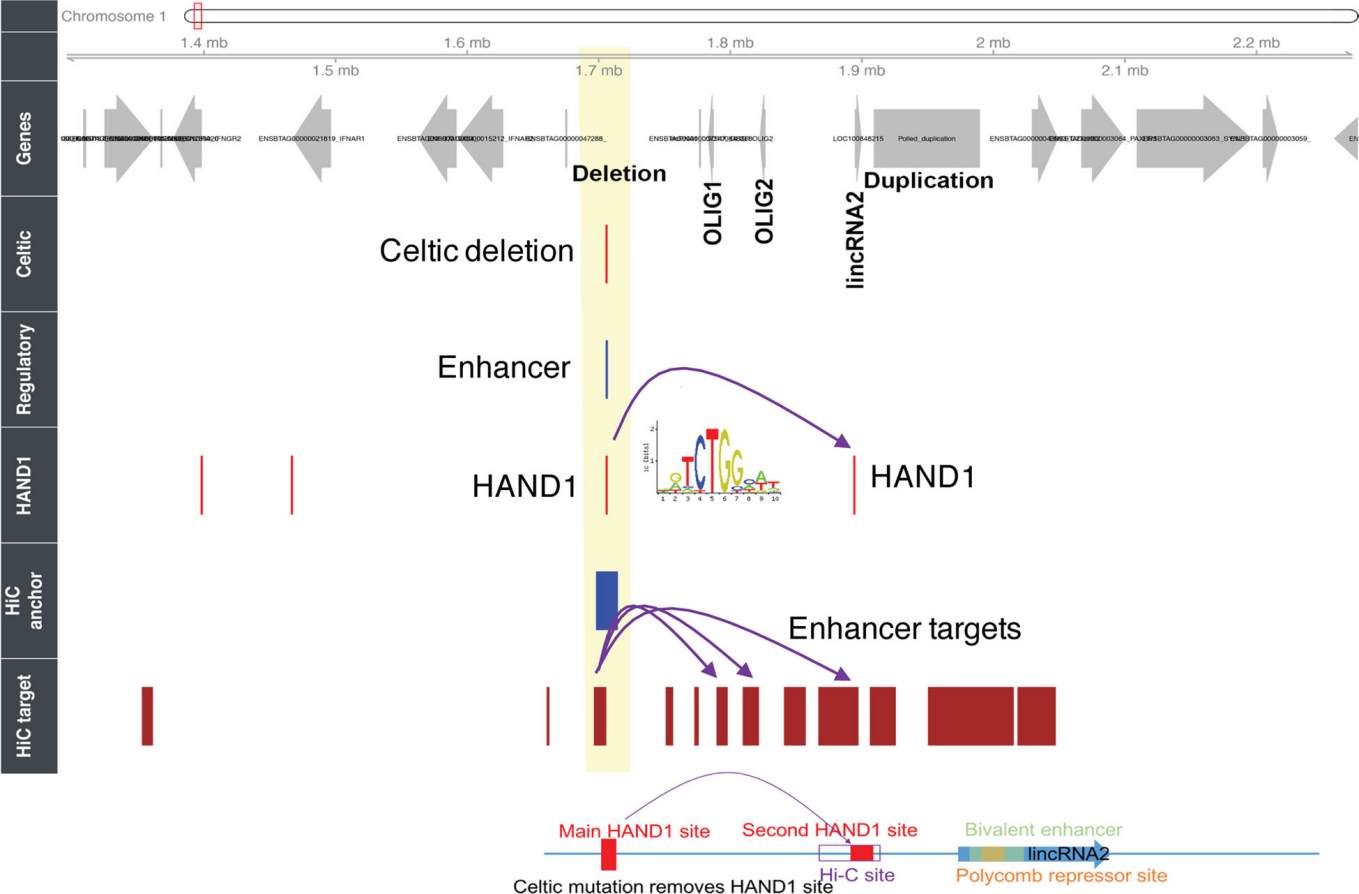
A potential model for effect of the Celtic mutation. Using human Hi-C (chromosome conformation capture) data and scanning of transcription factor binding sites, we generated a hypothesis to predict cattle regulatory targets for polled mutation (HiC target, HiC anchor, and HAND1 tracks). The regulatory (enhancers, promoters, and transcription factor binding sites) tracks display the HPRS-predicted region. Two common mutations on chromosome 1 in cattle have been associated with polled cattle. One is a 202-bp indel (“Celtic mutation”). The other is an 80-kb duplication ∼300 kb away. Purple arrows on the top link the Hi-C anchor to multiple targets mapped from the human genome to the cattle genome. Bottom of figure is a map with locations of the regulatory regions and shows potential effects of two HAND1 sites on the expression of the lincRNA2.

Another example of applying the HPRS databases for analysis of noncoding mutations is for the case of the “Celtic mutation,” which causes the polled phenotype. The mutation is a 202-bp indel, where the duplication of a 212-bp region (chr1: 1 705 834–1 706 045) replaces the 10 bp (chr1: 1 706 051–1 706 060) (Fig. 7) [46–48]. The mechanism for the Celtic mutation is unknown, although it may affect the expression of *OLIGO1, OLIGO2, CH1H21orf62*, and 2 long noncoding RNAs (*lincRNA1* and *lincRNA2*) [46, 47]. We found that the whole 10-base deletion, but not the upstream 212-base duplication, is within an HPRS-predicted enhancer sequence (chr1: 1 706 046–1 706 182, UMD3.1). A detailed transcription factor binding motif analysis of the polled mutation site suggests that a binding site for the Heart and neural crest derivatives expressed 1 (*TF HAND1*) is lost due to the 10-bp deletion in animals containing the Celtic mutation (Fig. 7C). The neural crest cells give rise to the craniofacial cartilage and bone [49], suggesting that the loss of the HAND1 putative binding site is a plausible explanation for the altered craniofacial development in polled animals. Additionally, using information from Hi-C in the human genome [44], we found the mutation is within a mapped interaction target of the regions Hi-C A_264 635 (chr1: 1 706 078–1 714 122, UMD3.1) and A_264 636 (chr1:1 698 252–1 706 077, UMD3.1) and interacts with genes hundreds of Kb away (Fig. 7, bottom panel; Table S2). Although the above hypothesis requires experimental validation, it shows that applying the HPRS approach could lead to a biological hypothesis for the underlying effects of causative mutations within noncoding regions.

Therefore, from the 2 examples described above (and from the Callipyge example described in the Supplementary Data), we found that the HPRS regulatory database can be used to prioritize SNPs and genetic variants that were identified by GWAS studies and to draw hypotheses about biological mechanisms of a causative SNP.

### Limitations of the methods

The main aim of the HPRS pipeline is to predict as many regulatory regions and as accurately as possible, so that the dataset could be applied for functional SNP analysis in the target species. However, given the uncertain nature of promoter and enhancer identification, the rate of false positives and negatives by HPRS is difficult to determine. In our analysis, all of the reference cattle liver enhancers were included in the initial unfiltered datasets, although ∼25% were lost during the filtering process. Similarly, 96% of reference cattle liver dataset promoters were covered by the unfiltered dataset, with less than 3% lost in the filtering process. A limitation of the HPRS filtering process is the requirement to use a species-specific dataset. Nevertheless, compared with the large number of datasets and biochemical assay types that are required to create comprehensive coverage of regulatory regions, the number of species-specific datasets needed for HPRS is small. In this paper, for each of the 3 species (mouse, cattle, and pig), we used data from only 3 biological replicates of the H3K27Ac assay, which was generated within a scope of 1 project, as reported in Villar et al. [22], to successfully filter the Universal Dataset. In addition, the approach cannot predict promoters and enhancers that are unique to the species, e.g., promoters and enhancers that are present in the cow but not present in humans. These unique promoters/enhancers are likely to be a small proportion of the total promoter/enhancer set. Indeed, the lineage-specific promoters and enhancers across 20 mammalian species were around 5% of the total promoters and enhancers [22]. Of note, relevant human input datasets can be integrated depending on the aim of an analysis. For example, if the focus is to study milk production, the HPRS pipeline can be applied for more relevant tissues, such as the mammary gland. Future cattle-specific datasets can be incorporated into the HPRS pipeline to address the tissue and species specificity issues.

In contrast to the HPRS pipeline prediction of regulatory regions, the prediction of causative genetic variation within regulatory regions is much more challenging. The current approach relies on the enrichment of sequence motifs within regulatory regions relative to nonregulatory regions. At least some of the motifs are TFBS, but there are likely to be other types of motifs, such as G-quadraplexes, present in regulatory regions. While the predicted datasets can be useful for generating relevant hypotheses, the identification of causal variants still requires considerable future refinement and validation.

## Conclusions

We have developed the HPRS pipeline using a large collection of existing human genomics data and a limited number of cattle-specific datasets to predict a database of cattle regulatory regions that covers a large number of active promoters, enhancers, and TFBS. The database generated here is not a final product because HPRS is capable of readily integrating new cattle-specific datasets into its mapping and filtering pipeline to expand, refine, and validate the databases. Moreover, the HPRS pipeline can be applied to data of other mammalian species and by scientists without computer programming skills. We anticipate that the pipeline will be used to integrate large-scale datasets from the FAANG consortium, when they become available, with complementary data from human research. The immediate application of the regulatory database is to complement the current species-specific GWAS analysis by (1) discovery of potential regulatory mechanisms of SNPs lying outside gene coding regions, (2) prioritizing SNPs that are statistically significant at a genome-wide level but located within regulatory regions, (3) prioritizing SNPs that are at low allele frequency but have potential for large effects, and (4) suggesting possible causative SNPs as targets for precise genome editing or selective breeding practices.

## Methods

The complete HPRS pipeline is divided into 3 modules: mapping, filtering, and SNP analysis. The whole pipeline and documentation are available from the CSIRO BitBucket [50].

### HPRS mapping pipeline

We developed a mapping strategy based on 4 elements: (1) selecting a suitable combination of human databases as HPRS inputs; (2) finding an optimal sequence identity threshold in the target genome; (3) finding options to remove less confident mapped results; and (4) adding multiple mapped regions that meet a high sequence similarity threshold. Depending on the species, targeted tissues, or regulatory categories of interest, users can select suitable human databases using the following suggested criteria: types of regulatory regions (promoters, enhancers, and TFBS), biochemical assays, computational models for combining data, and data sources (tissues, cell lines, traits). Second, by applying the UCSC liftOver tool [23], regions that were aligned at the genome scale (by LastZ pair-wise genome alignment [52]) were fine-mapped to identify target regions with proportion of sequence identity to the original regions (minMatch) higher than a selected cutoff. We recommend an optimal minMatch of 0.20 and not allowing multiple mapping for this step. Users can vary input parameters (minMatchMain and minMatchMulti) in the HPRS mapping script (Main_Mapping_Pipeline.py) to optimize the minMatch suitable to specific datasets that may have different features such as sequence length and conservation. Third, mapped regions resulting from using a low minMatch cutoff (0.20) were filtered to retain only regions with exact reciprocal mapping back to the human genome, with the condition that both the left and right borders of the reciprocally mapped regions were within 25-bp windows of the original regions. Fourth, to accommodate regions possibly resulting from duplication events, the HPRS mapping pipeline added a step to remap regions that are unmapped or are not reciprocally mapped by allowing multiple mapped results to be included while setting a high sequence similarity threshold (specified by the minMatchMulti parameter ≥0.80). Fig. S1A shows some of the expected mapping scenarios.

In addition to the customized minMatchMain and minMatchMulti parameter inputs, the Main_Mapping_Pipeline.py script also takes user-specified chain files for target species, which can be any of the mammalian species with chain files available from the UCSC databases or generated in-house. The HPRS mapping pipeline enables fast mapping of as many databases as necessary. The script PostHPRSMapping_MergeDifferentDatabaseTypes.py (available in the CSIRO BitBucket [50]) can be used to combine resulting datasets into 1 dataset containing nonoverlapping regions. For example, we merged enhancer databases from 88 ROADMAP tissues/primary cell lines and 5 additional promoter, enhancer, and TFBS databases. The script also collapses names of overlapping regions into a comma-separated field that can be used to count the total number of annotations for each merged region.

### HPRS filtering pipeline

Detailed description of the 7 filters is presented in the Supplementary Materials and Methods section. Briefly, the HPRS filtering pipeline was written in R and contains 7 filtering steps (Fig. 4, Table 4). The input file is a merged metadata file, in which each region was calculated for the number of CAGE peaks mapped, the RNA-Seq signal from 86 cattle RNA-Seq datasets, the Villar H3K27Ac signal, the SVM enhancer scores (enhancer activity predicted by a machine learning classification method, gkmSVM) [53], the number of overlapping annotations, the conservation score based on the UCSC 100-way vertebrate alignment [54], and the number of TFBS based on Cluster-Buster scanning [55]. The main filtering pipeline was HPRS_Filtering_pipeline.Rmd. We tested a range of parameters and recommend using the parameters set in the script. In addition, prior to running this main script, users can choose to optimize parameters suitable to specific datasets using the script HPRS_Filtering_optimize_FilterOrder.Rmd, which calculates Ratio_P_ and Ratio_E_ (average number of enhancers and promoters per Mb of the total length of all predicted enhancers and promoters) for each filter and for a range of filter parameters so that the optimal parameters are used in the main filtering pipeline. The filtering pipeline was written in a such a way that it is simple to add or remove filter layers depending on availability of species-specific data.

### Methods to apply HPRS dataset for regulatory SNP analysis

The HPRS dataset can be applied for the selection of top candidate SNPs in regulatory regions, which are present in existing genotyping SNP chips. The selected SNPs form a small set of SNPs that are more likely to be causal or associated to phenotypes. Using these SNPs for GWAS analysis may reduce noise compared with using a large number of SNPs that are noncausal but in high linkage disequilibrium to causal SNPs. The top candidate SNPs can be selected by the identification of SNPs belonging or not belonging to the following categories: the Universal Dataset, the Filtered Dataset, the TFBS of the predicted regulatory regions, and regulatory regions active in tissues related to the trait of interest. In addition, deltaSVM scores can be used as 1 of the indicators for potential SNP effects, as discussed in the Supplementary Methods section. Alternatively, the dataset can be used for post-GWAS analysis, in which significant SNPs in noncoding regions that are identified from GWAS can be assessed for potential effect on gene regulatory activity. We have discussed examples of applications for the cases of pleiotropic SNPs, climatic adaptation–associated SNPs, and associated SNPs’ milk-production traits (Fig. S1, Table S1), and of post-GWAS analysis for the stature phenotype and Callipyge phenotype (Fig. 6; Tables S2 and S3).

We developed an implementation pipeline of the gkm-SVM model to estimate SNP effects on enhancer activities in cattle by adapting the model to the case where very limited species-specific ChIP-Seq data are available for model training (Supplementary Materials and Methods).

### Data availability

We have made all HPRS Python and R scripts publically available with usage instruction from the CSIRO BitBucket [50]. These codes can be used to perform all steps from mapping to filtering and scoring regulatory SNPs. Supporting data are also available via the *GigaScience* database, *Giga*DB [51].

All human databases used for prediction are publically available (Table S5). Results of predicted regulatory regions, including the Universal Datasets and the Filtered Datasets for cattle and pig, are available in the Supplementary Data for this article. For cattle, we provide deltaSVM scores for ∼97 million SNPs, which can be used as 1 of the parameters for assessing potential SNP effects. Additionally, we share predicted Universal Datasets (not yet filtered) for 10 other mammalian species in a format compatible for uploading to the UCSC genome browser (Table 5; Fig. S7). These 10 additional datasets can be useful for exploring potential regulatory effects from noncoding genomic regions.

## Additional files

Additional file 1: Figure S1: Assessing mapping results for different human tissues and cell line datasets onto other species. The counts and percentages are for mapped regions before the HPRS filtering pipeline. A, Possible cross-species mapping results, with 3 scenarios: (i) reciprocal mapping with a low identity threshold (minMatch = 0.20) but that requires exact back mapping to the human genome; (ii) multiple mapping allowing multiple targets, but requiring a stringent minMatch = 0.80; and (iii) features that do not fall into the 2 categories above, such as species-specific enhancers, are not included in the prediction. B, Coverage of the cattle Villar enhancer reference dataset by predicted and random feature datasets. Features were mapped from 42 human enhancer datasets or 42 random datasets (equal number of regions and region length distribution) to the bovine genome and then compared for percent overlap with the cattle Villar reference liver enhancer dataset. For all 42 datasets, the regulatory datasets produced 5–10 times higher coverage of the reference bovine liver enhancers than the random datasets. C, Coverage of predictions by 12 common ENCODE human cell lines (x-axis). The numbers of recovered regions using minMatch = 0.2 and minMatch = 0.95 are shown. D, Percentage of additional features that had multiple mapped targets but met the high sequence similarity threshold for reciprocal mapping from the bovine genome to the human genome (minMatch = 0.80).

Additional file 2: Figure S2: Enrichment of GWAS-associated SNPs in HPRS filtered regulatory regions. Data are from a GWAS dataset for 2112 cattle measured for 10 different climatic change adaptation–related phenotypes [6]. A, Number of GWAS significant SNPs, with –log(*P*-values) ≥7 in any of the 10 separate phenotypes. GWAS *P*-values for each trait ≤10^-7^ are considered significant with multiple test correction (with Bonferroni-corrected *P* ≤0.05, and the number of SNP is ∼500 000 SNPs). SNPs were selected for each of the 10 phenotypes separately and were pooled into 1 set, and density plots of SNP counts and corresponding –log(*P*-values) for each phenotype are shown. The x-axis shows –log(*P*-values) values from 0 to 20, and the y-axis shows density of the SNP counts according to the –log*P* distribution. B, Significant SNPS were selected based on combined criteria: –log(*P*-values) >2 and abs(effect size) greater than or equal to the third quartile effect size value for each of the 10 phenotypes. The x-axis shows the name IDs of the 10 phenotypes.

Additional file 3: Figure S3: Promoter prediction. A, We selected a random large region of the chromosome to evaluate promoter prediction. We observed consistent overlapping of predicted promoters with known transcription start sites (TSS). The higher and denser numbers of predicted promoters compared with annotated TSS suggest that the HPRS prediction potentially led to the identification of unannotated promoters, including alternative promoters within annotated transcripts and promoters of unannotated transcripts such as those for long noncoding RNAs. B, HPRS promoters also predict bidirectional promoters with high accuracy (- for antisense, + for sense). C, HPRS-predicted alternative promoters are supported by cattle expression sequencing tag (EST) data. The predicted promoters overlap the start sites of EST transcripts within the full-length ZC3H14 gene.

Additional file 4: Figure S4: Enrichment of TFBS within enhancers and promoters. The promoters and enhancers were mapped from the human FANTOM enhancer [1] and the human FANTOM promoter databases onto the bovine genome [2]. Mapped regions were compared with the Villar bovine enhancer and promoter reference datasets for liver tissues [7]. Three categories of overlapping to the reference datasets were compared (x-axis): (i) mapped regions overlapping the Villar reference dataset (LOinLiver); (ii) mapped regions not in the Villar dataset (LOnotinLiver); and (iii) regions in reference datasets not covered by mapped regions (LiverNotinLO). The TFBS were derived from whole–bovine genome scanning using the Cluster Buster program [13] and 3 major transcription factor position weight matrix databases (TRANSFAC, JASPAR, and ENCODE) [21–23].

Additional file 5: Figure S5: Tissue specificity of predicted regulatory regions. A, Counts of HPRS-predicted enhancers (using 88 ROADMAP human enhancer datasets) that overlap with the Villar cattle reference enhancers. B, We then defined a tissue-specific enhancer dataset by identifying HPRS regions that overlap with Villar reference enhancers for cattle and are unique to each of the 88 tissues. The datasets that yielded the highest overlap are those from the liver cell line (liver hepatocellular cells - HepG2) and the human liver tissue. C, We mapped 101 RNA sequencing datasets, collected from more than 79 tissues (Table S5), to the predicted regulatory regions. The mapped RNA signal was used to compare the similarity between different tissues. Strong enrichment of brain, muscle, and liver tissues was observed.

Additional file 6: Figure S6: Large-scale gapped k-mer support vector machine (LS-gkm-SVM) scores for enhancers and deltaSVM scores for SNPs. A, The LS-gkm-SVM model was used to calculate the gkm-SVM scores for all enhancers in the Villar dataset. Red, enhancers scored on “enhancers versus background matrix”; green, random regions (selected by shuffling through the genomes to sample genomic regions of the same length as the Villar reference bovine enhancers) scored on “enhancers versus background matrix”; blue, enhancers scored on a “background versus background” matrix. The positive background was selected from the Villar reference enhancer dataset, as described in the Supplementary Materials and Methods section. Training datasets using human (HHb) and cattle (BBb) and liftOver enhancer regions from human to cattle (LOBHb) yielded consistent and comparable results, which predicted higher scores for enhancer regions (BBb_Enh, HHb_Enh, LOBHb_Enh) than the predictions for promoter (pmtr_Enh) and random regions (BBb_Neg, HHb_Neg, LOBHb_Neg, and Pmtr_neg). B, deltaSVM for scoring SNP effects on enhancer activity. The LS-gkm-SVM model was used to score every possible SNP across the enhancer of the aldolase B fructose bisphosphate (*ALDOB*) gene in cattle. Single nucleotide resolution scores within the *ALDOB* enhancer are shown. Negative scores indicate loss of function (or TF binding), while positive scores indicate increases in activities. Computational predictions of transcription factor binding sites (by FIMO [29] and JASPAR position weight matrices) are shown in the lower panels. Transcription factor IDs and SNP IDs are shown next to the predicted regions. The *ALDOB* enhancer was mapped from humans to cattle. Vertical dashed lines show the locations of the deltaSVM peaks, where SNPs most likely reduce the enhancer activity, compared with the locations of predicted TFBS. The deltaSVM score prediction was consistent with luciferase activity measurement (in humans) and prediction of TFBS (in humans and cattle).

Additional file 7: Figure S7: An example of a simple view of the datasets generated for 10 mammalian species. The example is from the dog (canFam3) genome. Predicted regulatory regions are shown in blue, with annotations (enhancer, promoter, and transcription factor IDs) marked on the left. For regions with multiple annotations, users can display the annotations by selecting the region on the browser. The example shows the *ENPP1* gene.

Additional file 8: Table S1: Enrichment of GWAS SNPs in common phenotypes measured for dairy cattle.

Additional file 9: Table S2: Hi-C targets and gkm-SVM predict causative SNPs and gene targets.

Additional file 10: Table S3: Predicted Hi-C interaction regions at the putative Callipyge locus (chr21:67339968:67340027).

Additional file 11: Table S4: Regulatory datasets used for optimizing mapping parameters.

Additional file 12: Table S5: Data sources for publically available human datasets used as inputs for the HPRA pipeline in this paper.

## Abbreviations

bp: base pair; CAGE: Cap Analysis of Gene Expression; CDS: coding sequence; ENCODE: Encyclopedia of DNA Elements; FANTOM: Functional Annotation of the Mammalian genome; FD: Filtered Dataset; Gb: Giga base; GWAS: Genome-Wide Association Studies; HPRS: Human Projection of Regulatory Regions; kb: kilo base; Mb: Mega base pair; RDEs: Regulatory DNA Elements; ROADMAP: Roadmap Epigenomics Mapping Consortium; SNP: Single Nucleotide Polymorphism; SRA: Sequence Read Archive; SVM: Support Vector Machine; TE: Transposable Elements; TFs: Transcription Factors; UCSC: University of California Santa Cruz; ENSEMBL: Ensemnbl database; TSS: Transcription Start Sites; TFBS: Transcription Binding Sites; PWM: Position Weight Matrix; UD: Universal Dataset.

## Competing interests

The authors declare that they have no competing interests.

## Funding

Q.N. and M.N.S. were supported by CSIRO OCE Postdoctoral Fellowships.

## Author contributions

B.D., R.T., J.K., B.B., and Q.N. conceived the project. Q.N. and B.D. designed the HPRS algorithm. Q.N. wrote the pipeline. Q.N., M.N.S., L.P.N., A.R., and B.H. contributed to the analysis. Q.N. and B.D. wrote the manuscript, with input from all other co-authors.

## Supplementary Material

GIGA-D-17-00310_Original_Submission.pdf

GIGA-D-17-00310_Revision_1.pdf

Response_to_Reviewer_Comments_Original_Submission.pdf

Reviewer_1_Report_(Original_Submission) -- Christiaan Henkel 04 Dec 2017 Reviewed

Reviewer_2_Report_(Original_Submission) -- Ole K Tørresen 06 Dec 2017 Reviewed

Supplemental material
