# Supplementary material for: Mammalian genomic regulatory regions predicted by utilizing human genomics, transcriptomics, and epigenetics data
Source: Gigascience. 2018 Feb 16;7(3):gix136. doi: 10.1093/gigascience/gix136 (PMC5838836; doi:10.1093/gigascience/gix136)
Supplement: Supplemental material [file gix136_supp.pdf]

Mammalian genomic regulatory regions predicted by utilizing human genomics, transcriptomics and epigenetics data

Quan H. Nguyen[1,2], Ross L. Tellam[1], Marina Naval-Sanchez[1], Laercio R. Porto-Neto[1], William Barendse[3], Antonio Reverter[1], Benjamin Hayes[4], James Kijas[1], and Brian P. Dalrymple[1,5*]

Affiliations:

[1] CSIRO Agriculture, 306 Carmody Road, St. Lucia, 4067, QLD, Australia

[2] Divisions of Genomics of Development and Disease, Institute for Molecular Bioscience, University of Queensland, 306 Carmody Road, St. Lucia, 4067, QLD, Australia

[3]School of Veterinary Science, University of Queensland, Gatton, 4343, QLD, Australia

[4]The Queensland Alliance for Agriculture and Food Innovation (QAAFI), University of Queensland, 4067, QLD, Australia

[5]Institute of Agriculture, The University of Western Australia, Perth, Western Australia, 6009, Australia

[*]Correspondence: Brian P. Dalrymple (brian.dalrymple@uwa.edu.au)

Optimizing parameters for mapping of regulatory regions

To identify putative regulatory regions possibly generated by duplication events in the bovine lineage (see Fig. S1a for mapping scenarios), the HPRS mapping pipeline pooled unmapped regions in the human datasets (with minMatch = 0.2) and mapped regions with no exact reciprocal matches (from minMatch = 0.2), for a second round of mapping with different parameters to rescue regions with multiple mapped targets. For these regions, we applied liftOver from human to the targeted species with two parameters: 1) allowing multiple mapped results; and 2) keeping only results that passed a high sequence similarity threshold ($\geq$ 0.80). We assessed the percent of regions rescued from this additional step by testing 88 ROADMAP enhancer datasets (Fig. S1d, Table S8). Across the 88 datasets (Table S8), this additional multiple-map process rescued, on average, 11.9% of the total predicted regulatory regions for each dataset.

Next we asked whether the regions identified with the selected parameters applied to a human enhancer dataset outperformed a random set of regions sampled from the bovine genome. We randomly sampled the whole bovine genome sequence to generate 42 independent sets of random sequences with equal numbers and length distributions to the sequences in each of the 42 human ROADMAP datasets (38 adult tissues and four cell lines/cell cultures) (Fig. S1b). Consistently across the 42 tissues/cell lines, the ROADMAP enhancers mapped to the Villar reference cattle dataset 2.5 to 4.5-fold more frequently than the random datasets. The minMatch parameter of 0.2 also performed better (5-10 times higher) than 0.95 for mapping 12 enhancer datasets from 12 different cell lines from the ENCODE project to the Villar reference cattle liver enhancer dataset (Fig. S1c). Taken together, this approach identified an optimised set of mapping parameters for the projection of regulatory sequences in humans onto the bovine genome. Next, we developed a strategy to capture most regulatory regions across different tissues, conditions, and regulatory categories.

Transcription factor binding site analysis

We asked if the mapped TFBSs from ENCODE datasets to cattle had more overlapping regions than random when comparing against two independently derived feature sets from de novo motif prediction based on cattle-specific DNA sequence. We made use of the Bickhart TFBS dataset, which predicted TFBSs upstream of 8,000 cattle genes, focusing on TFBSs at promoter regions [1]. We observed that 236,997 (79.4%) TFBS enriched regions predicted by Bickhart et al. [1] overlapped the HPRS mapped ENCODE proximal TFBS sites. We performed 100 bootstrap randomizations to sample 100 random datasets, each containing the same number of regions (377,607) to the Bickhart et al. dataset, and each sequence had the same length to the corresponding sequence in the Bickhart et al. dataset. The 95 percentile of the random overlap with the 298,554 proximal TFBSs was 7,274 regions, displaying a 32.5 times lower coverage than the overlap using the Bickhart et al. dataset. The high agreement between the HPRS mapped ENCODE TFBSs and the Bickhart et al. predicted TFBSs suggests that the predicted regions are likely represent real TFBSs and that non-overlapping regions of the HPRS predicted by proximal TFs may be a large expansion to the Bickhart dataset, which was designed for a smaller scale of the genome (i.e. 8000 upstream regions). Second, we applied the Cluster-Buster (CB) program [2], to scan for all possible binding sites based on bovine DNA sequence and known conserved transcription factor binding position weight matrices, an approach independent of prior knowledge of gene annotation, different to the approach used by Bickhart et al. CB was run separately for each PWM taken from three transcription factor databases TRANSFAC, JASPAR, and ENCODE [3-5] and scanned the whole bovine genome for possible binding site of each TF. The CB results supported the HPRS predicted distal TFBS dataset. Whilst 433,478 out of 749,572 overlapped with CB-TFBS enriched regions, the 95 percentile of the random overlap (with 100 randomization as described above) was 204,087 regions (Fisher's exact test $p < 2.2e-16$; 95% C.I. odds ratio 3.64-3.69).

More significant SNPs within regulatory regions

Significant SNPs associated with 10 different cattle climatic adaptation related traits in 2,112 Brahman cattle, genotyped by BovineSNP50 (imputed to 770K) and 770 K BovineHD chips were identified in a published GWAS study [6]. The 10 phenotypes included yearling weight (YWT), flight time (FT), rectal temperature (TEMP), penile sheath score (SHEATH), coat colour (COLOUR), coat score (COAT), tick infestation (TICK), fly lesions (FLY), condition score (COND), and endoparasite eggs per gram measured in faeces (EPG). SNPs were placed into two categories i.e. in a filtered regulatory region and not in a filtered regulatory region. The number of significantly associated SNPs from the GWAS analyses that were within the HPRS filtered regulatory regions was higher compared to the number of significantly associated SNPs within non-regulatory regions across most phenotypes (Fig. S2). We also observed enrichment of GWAS SNPs in predicted regulatory regions for common phenotypes measured for dairy cattle (Table S1).

Predict potentially novel mutation

The callipyge mutation in sheep is a change from A to G in the intergenic imprinted region on chromosome 18 between the genes *DLK1* and *MEG3* [7]. When the mutation is paternally inherited in a heterozygote the sheep postnatally develop hypertrophy in specific skeletal muscles positioned toward the rear of the animal. The paternal inheritance of the mutation in the context of the heterozygote genotype is also associated with striking upregulation of the expression of *DLK1* and *RTL1*, two paternally expressed genes in the imprinted region, and to a lesser extent *MEG3,* a maternally expressed gene, and a number of miRNAs [8-10]. The HPRS pipeline identified an enhancer across the region of the bovine genome orthologous to the region containing the sheep callipyge mutation. In this region, the two species have 100% sequence identity (chr21:67,339,968-67,340,027 in cattle UMD3.1).

The predicted enhancer in cattle that overlaps the callipyge mutation is consistently detected in all 88 tissues of the ROADMAP enhancer dataset. However, the deltaSVM score for this mutation in cattle is not significant (Tables S2, S3). Although the SNP lies in a predicted MyoD binding site, the SNP did not change the binding affinity of MyoD/E47 [11]. Based on the human Hi-C data the callipyge mutation lies in the potential Hi-C target of 21 anchors and these anchors are positioned adjacent to multiple genes in the imprinted region, including *DLK1*, *RTL1*, *bta-mir-431* and *MEG3* (Table S2), many of which are normally coordinately regulated and their expression changes considerably in the callipyge paternal heterozygote. Thus, the site of the mutation is predicted to be in an enhancer (or imprinting boundary element) that regulates the expression of several genes in this imprinted locus. Further studies are required to dissect the differing influences of the mutation in the context of the other two genotypes (the maternal heterozygote and the homozygous genotype), which do not generate postnatal muscle hypertrophy.

Selection of seven filters for the filtering pipeline

The optimization of dataset selection and of the mapping strategy is described in the main text. We present in this section details of the filtering component of the HPRS pipeline, which takes input from the HPRS mapping output. The filtering component was designed to incorporate the specificity of the target genome DNA sequence to predict a high confidence set of probable functional elements (the filtered dataset). Seven data types were used to filter the putative regulatory regions: number of CAGE peaks; H3K27Ac signal; RNAseq signal; gkmSVM scores; total annotation counts; phastCons scores, and; total number of TFBSs. The pipeline was executed in a hierarchical order. After each filter, the set of regions that met the filtering criteria was added to the final filtered dataset, while all remaining regions not passing the criteria were subjected to the next filtering step. The 7 filters were ordered from the top (the filter that produced the highest coverage of reference enhancers and promoters and the highest

$Ratio_E$ and $Ratio_P$-See method section in the main text for $Ratio_E$ and $Ratio_P$) to the bottom (with lower coverage and lower $Ratio_E$ and $Ratio_P$) of the hierarchical pipeline.

We used CAGE as one of the filtering criteria because bidirectional human CAGE peaks are strong marks for active enhancers [12] and CAGE data has a powerful ability to identify transcription start sites of the majority of transcripts [13]. The HPRS pipeline maps all available human CAGE peaks from the FANTOM5 human promoter atlas [13] to the target species genome. The number of mapped peaks overlapping each of the predicted regulatory regions was counted and the normalized counts of CAGE peaks (per bp) per regulatory region was used as a HPRS filter parameter.

We included H3K27Ac and RNA-Seq as two of the seven filters. Not only is RNA-Seq data a signal of active transcription, it may also mark the presence of enhancer RNA (eRNA), which is a signal for active enhancers [14, 15]. Similarly, the H3K27Ac data is among the strongest signature for active enhancers while also marking active promoters [14, 16]. To apply these two filters, cattle-specific RNA-Seq data from 79 tissues [17] and four H3K27Ac ChIP-Seq datasets from cattle liver tissues [18] were mapped to the bovine genome (UMD3.1) using BWA mapping [19] with FASTQ files as inputs. The output BAM files were then used for calculating the normalized RPKM (Reads Per Kilobase of transcript per Million mapped reads) signal using featureCounts [20].

Next we added a novel filtering criterion using a recent machine learning classification method, gkmSVM [21, 22]. The detailed implementation of the gkmSVM is described in the next section.

Another useful feature to prioritize functional genomic regions is the overlap of multiple functional categories, for example one region containing binding sites for a number of different transcription factors may be classified as a promoter and as an enhancer. The HPRS

filter uses one layer to utilize this feature by calculating the number of different annotations for each of the predicted regulatory regions. The higher the number of independent datasets that predicted the same regulatory region, the greater the chance of it being a real functional region. The overlapping annotation includes: the number of human tissues in the ROADMAP database; the number of CAGE promoters and enhancers, and; the number of mapped ENCODE distal and proximal TFBSs. The Universal Dataset from the HPRS mapping consists of merged regions, where coordinates and annotations of overlapping regions were combined into a common region. We used annotation count as one of the filtering criteria, and found that this filter was especially powerful to enrich for promoters as it could increase the density of promoters by ~26-45 fold and 9-12 fold compared to the genome and the universal base lines, respectively. The density increase was relatively lower for enhancers.

Additionally, we utilized sequence conservation of functional regions across species to filter for highly conserved regulatory regions. Active enhancers in human, defined by CAGE, have been shown to have higher phastCons scores than random regions [12]. We calculated the phastCons conservation score for each region based on the UCSC 100-way Vertebrate Alignment [23]. Applying the PhastCons filter increased the promoter density by ~10 fold compared to the genome base line (**Table 3**).

The consensus binding sequences of many transcription factors (TFs) are known and the position weight matrices (PWMs) of their binding sites can be readily mapped to the target species genome. The de novo prediction of transcription factor binding sites (TFBSs), by Cluster-Buster scanning for example [2], can be used for identifying promoters and enhancers (Fig. S4). However, the identification of TFBSs based on sequence alone has very limited predictive power for functional TFBSs in the absence of other information about the region being analysed e.g. the chromatin context. In a prediction of TFBSs in the bovine genome, regions upstream of ~8,000 genes likely to be promoters based on location were analysed [1].

Thus, in this instance TFBSs were not defining regulatory regions, rather regulatory regions needed to be defined to allow functional TFBSs to be predicted. Since our objective was to use TFBS data to help define regulatory regions, a different approach was required. We predicted potential regulatory regions first, then used de novo scanned sites at the genome wide level to score these predicted regions. We applied the Cluster-Buster (CB) program [2] to scan for all possible binding sites, independent of gene annotation. This generated 1,131,366 TFBSs, spanning 563.814 Mb. The TFBS filter increased promoter density by 62-79 times compared to the genome base line, but less for enhancers.

Implementation of the machine learning classification method gkmSVM

gkmSVM was implemented to score regulatory activity in cattle for each of the predicted regulatory regions in the universal dataset (all the raw mapped regions to the bovine genome). The gkmSVM model uses a positive training set (e.g. known enhancers) and a set of negative regions (random segments of the genome with similar nucleotide characteristics but without H3K27Ac enhancer signal) to build a model based on sequences over-represented in the positive set. The model estimates the enhancer enrichment scores for every possible 10 bp sequence, which can be combined to calculate enhancer activity scores for regions of interest. gkmSVM often requires large training datasets. We developed an approach to building highly contrasting positive and negative training matrices from a small number of cattle H3K27Ac datasets (see Python codes at https://bitbucket.csiro.au/users/ngu121/repos/hprs/browse/RegulatorySNP/Implementing_gkmSVM/). We generated positive regulatory training datasets based on all H3K27Ac peaks [18]. H3K27ac marks active promoters of genes and active enhancers [16]. From the MACS2 summit file, peaks were extended 50 bp 5’ and 3’ of the centre point. The resulting regions were pooled into one file and overlapping regions were merged to form the final set of positive regulatory regions. The script Prepare_Pos_Enh_MultiSummits.py can be used for selecting

peaks from any MACS2 output file. Next, a negative training set was created based on the positive set using the script Prepare_Neg_3xPosSize.py. We randomly shuffled the whole genome to select a training set with length and repeat content similar to the positive set and we increased the number of negative regions to three times more regions than the positive training set [22]. The negative set was selected in a way that only regions with no H3K27Ac signal were kept (by running the script Filter_NegativeSet_byH3K27AcCount.py). Any sequences with ambiguous base calling Ns were removed using the script RemoveSequences_with_N.py. To train these processed datasets, we used the LS-gkm-SVM software version, which takes large input files [21], with a modification in the LS-gkm-SVM script to increase the maximum size of the input region to 5,000 bp. The approach generated high contrast training matrices, which can improve the differentiation power of gkm-SVM scores to distinguish the positive reference enhancer dataset from a randomly selected set of sequences (Fig. S6), and therefore we used gkm-SVM scores as one of the criteria for the HPRS filter pipeline. The gkm-SVM filter increased the density of promoters and enhancers (per Mb) by ~4 times and ~10 times compared to the genome base lines, respectively (Table 3).

Scoring SNPs for predicted causative effect

The gkm-SVM program scores activity of each individual regulatory region and can also be used to score the potential effect of individual SNPs on the activity of an enhancer [21, 22]. DeltaSVM scores are estimates of activity differences of sequences containing one allele of a SNP versus another allele of the same SNP. We developed an approach to building highly contrasting positive and negative training matrices from a small number of cattle H3K27Ac datasets (see Python codes in the HPRS repository https://bitbucket.csiro.au/users/ngu121/repos/hprs/). We then scored ~ 97 million SNPs from the cattle dbSNP database (dbSNP build 146). Each SNP was centred in a 300 bp window and we calculated deltaSVM scores by comparing sequences with and without the SNP. We tested

deltaSVM scores in a predicted *ALDOB* enhancer in cattle [22, 24]. DeltaSVM scores for this enhancer decreased markedly at locations overlapping transcription factor binding sites, indicating reduction or loss of binding for one allele versus the other) (Fig. S6b). The deltaSVM scores of SNPs provide an additional parameter for prioritizing genome-wide significant SNPs identified by GWAS analysis, as discussed in the next section.

Other computational and statistical analysis

In addition to the HPRS Python and R scripts (available at https://bitbucket.csiro.au/users/ngu121/repos/hprs/browse), several other open-source programs were used, including LS-gkm-SVM [21], Cluster-Buster [2], Bedtools/2.18 (intersect, shuffle, groupby, map and merge utilities) [25], featureCounts [20], BWA/0.7.12 [19], SAMtools/1.2.1 [26], and GNU parallel (by Ole Tange). Figures in the paper were produced by using the following R packages: ggplot2, ggbio, Gviz, VENN, VennDiagram, GenomicFeature, plyr and stringr.

Randomisation tests were performed by bootstrap sampling 100 times, which was used to construct a null distribution. Random sampling of a genomic region was performed using the bedtools *shuffle* utility. The observed value was then compared to the 5 and 95 percentile. Fisher's exact test and the hypergeometric test were applied to calculate enrichment over random regions.

Supplementary Tables

Table S1. Enrichment of GWAS SNPs in common phenotypes measured for dairy cattle.

		Random distributions		
	Observed significant SNPs	5th percentile	Median	95th percentile
Fat kg	1132	1032	1091	1142
Milk kg	1682*[1]	1526	1592	1650
Fertility (calving interval)	47*	25	35	45
Somatic cell count	91	85	98	114
Protein kg	1809*	1641	1707	1793

The GWAS data was taken from a study for 17,925 Holstein and Jersey cattle genotyped for 632,003 SNPs, on 5 major production and functional traits in dairy cattle (Raven et al., 2014, BMC genomics**, 15**:62). The number of significant GWAS SNPs (*P-value* < 0.0001) that are shared with the set of 37,657 SNPs in the genotyped set of 632,003 SNPs and within regulatory regions are shown in the observed significant SNPs column. An equivalent number of randomly chosen SNPs (37,657) from the HD chip (but present in the regulatory SNP set) were used to ascertain how many were significant by chance (*P-value* < 0.0001). For each trait, 100 randomizations were used to construct the null distribution.

[1]* significantly more SNPs than expected by chance at *P-value* < 0.05. given the null distribution.

Table S2. Hi-C targets and gkm-SVM predict causative SNPs and gene targets

Region	SNP/variant	Variant type	Coordinate	Regulatory region	gkm-SVM score for regulatory region[1]	Delta-SVM score for SNP/variant	Hi-C in human genome	Hi-C target liftover bovine genome	Nearest genes to Hi-C target(s) (<2Kb)
PLAG1	rs109815800	G/T	chr14:25015640	Enhancer (chr14:25014141-25016706)	-2.6	3.2	A_447037 (chr14:25015628-25020227)	Target was not mapped	NA
PLAG1	rs209821678	(CCG)x11 /(CCG)x9	chr14:25052396	Promoter (chr14:25052350-25052511)	1.9	-6.0	A_447043 (chr14:25044319-25054287)	chr14:25478861-25497096	*IMPAD1*
PLAG1	rs210030313	A/G	chr14:25052440	Promoter (chr14:25052350-25052511)	1.9	0.51	A_447043 (chr14:25044319-25054287)	chr14:25478861-25497096	*IMPAD1*
Callipyge	Not yet reported in cattle	A/G (in sheep)	chr21:67339968-67340027 (mapped from sheep)	Enhancer (chr21:67315310-67342921)	3.0	1.5	Multiple overlapping anchors (see Table S3)	Targets of 21 anchors (see Table S3)	*DLK1* (Delta-Like 1 Homolog), *RTL1*, *MIR431*, *MEG3*
Poll Celtic mutation	212 bp duplication	indel	chr1:1705834-1706045	no	NA	NA	no	no	NA
Poll Celtic mutation	10 bp deletion	indel	chr1:1706051-1706060	Enhancer (chr1:1706046-1706182)	0.02	5.2	A_264635 and A_264636	14 targets (Table S3, Fig. 7)	lincRNA1, lincRNA2, OLIG2, OLIG1, C21orf6, TMEM50B

[1]A SNP with a high absolute value of gkm-SVM score suggests that the SNP is likely to have regulatory effect. The range of gkm-SVM scores for all 97 million SNPS is used as the bovine genome-wide reference distribution of gkm-SVM scores. The scores of SNPs of interest can be compared to the reference distribution to assess the likelihood of the SNP being functional.

Table S3. Predicted Hi-C interaction regions at the putative Callipyge locus (chr21:67339968:67340027).

Chr_Target[1]	Start_Target	End_Target	Anchor_ID[2]	Chr_Anchor	Start_Anchor	End_Anchor	Gene_ID	Distance to gene
chr21	67280798	67411436	A_144785	chr21	67270155	67280797	ENSBTAG00000037899_DLK1	0
chr21	67297318	67380553	A_144778	chr21	67217803	67234249	ENSBTAG00000037899_DLK1	14230
chr21	67313630	67343047	A_144786	chr21	67280798	67289513	ENSBTAG00000037899_DLK1	30542
chr21	67314901	67343047	A_144792	chr21	67312744	67314699	ENSBTAG00000037899_DLK1	31813
chr21	67314901	67401626	A_144773	chr21	67197780	67201840	ENSBTAG00000029792 (ovis-MIR493)	14970
chr21	67322329	67343047	A_144787	Not mapped	Not mapped	Not mapped	ENSBTAG00000037899_DLK1	39241
chr21	67322329	67343047	A_144790	Not mapped	Not mapped	Not mapped	ENSBTAG00000037899_DLK1	39241
chr21	67335996	67343047	A_144793	chr21	67314700	67322328	ENSBTAG00000037899_DLK1	52908
chr21	67335996	67361921	A_144747	chr21	67003202	67004893	ENSBTAG00000037899_DLK1	52908
chr21	67335996	67361921	A_144853	chr21	67760692	67764115	ENSBTAG00000037899_DLK1	52908
chr21	67335996	67380553	A_144737	chr21	66954647	66957938	ENSBTAG00000029792 (ovis-MIR493)	36043
chr21	67338105	67343047	A_144794	chr21	67322329	67335995	ENSBTAG00000037899_DLK1	55017
chr21	67338105	67343047	A_144797	chr21	67343201	67361921	ENSBTAG00000037899_DLK1	55017
chr21	67338105	67343047	A_144799	chr21	67379704	67386004	ENSBTAG00000037899_DLK1	55017
chr21	67338105	67343047	A_144801	chr21	67393596	67400924	ENSBTAG00000037899_DLK1	55017
chr21	67338105	67343047	A_144804	chr21	67432764	67442691	ENSBTAG00000037899_DLK1	55017
chr21	67338105	67379703	A_144791	Not mapped	Not mapped	Not mapped	ENSBTAG00000029792 (ovis-MIR493)	36893
chr21	67338105	67379703	A_144791	Not mapped	Not mapped	Not mapped	MEG3	0
chr21	67338105	67380553	A_144782	chr21	67246561	67256982	ENSBTAG00000029792 (ovis-MIR493)	36043

chr21	67338105	67393596	A_144738	chr21	66957939	66967243	ENSBTAG00000029792 (ovis-MIR493)	23000
chr21	67338105	67400924	A_144736	Not mapped	Not mapped	Not mapped	ENSBTAG00000029792 (ovis-MIR493)	15672
chr21	67338105	67434783	A_144728	chr21	66929297	66931499	ENSBTAG00000029792 (ovis-MIR493)	0
chr21	67338105	67434783	A_144728	chr21	66929297	66931499	ENSBTAG00000037265 (ovis-MIR665)	0
chr21	67338105	67434783	A_144728	chr21	66929297	66931499	ENSBTAG00000046585_RTL1	0
chr21	67338105	67434783	A_144728	chr21	66929297	66931499	ENSBTAG00000030002 (bta-MIR431)	0

[1, 2]In the all-to-all capture methods (Hi-C and ChiA-PET) context, the labels ‘target’ and ‘anchor’ can be used interchangeably to refer to two distant DNA fragments looping in three dimensional space to come to contact.

Table S4. Regulatory datasets used for optimizing mapping parameters.

Dataset	Number regions	Region types	Mean length (bp)	Tissues/cell lines	Data types[1]	Models[2]
ENCODE	108000	Enhancers	674.9	0/3	ChIP-Seq	hiHMM
ROADMAP	113839	Enhancers	1119.6	Liver/0	ChIP-Seq	chromHMM
FANTOM Enhancers	43011	Enhancers	289	135/673	CAGE	Bidirectional CAGE
ENSEMBL	2427934	Enhancer	548.5	0/18	ChIP-Seq, DNAse I	Segway
Vista (activity validated)	1740	Enhancers	1780.2	>23	Reporter assay	Highly conserved
FANTOM Promoters	201802	Promoters	21.5	152/823	CAGE	DPI
Exon	742293	Exons	299.9	na/na	UCSC hg19 known gene	UCSC annotation

[1]ChIP-Seq: chromatin immunoprecipitation sequencing; CAGE: Capped analysis of gene expression; DNAse I: sequencing for detecting DNAse I hypersensitive sites.

[2]HiHMM: hierarchically linked infinite hidden Markov model; chromHMM: Chromatin state HMM; DPI: decomposition-based peak identification.

Table S5. Data sources for publically available human datasets used as inputs for the HPRA pipeline in this paper.

Database	Description	URLs	Date accessed
ENCODE TF peaks (version 2)	All ENCODE proximal and distal TFs produced by combining original ENCODE TF ChiP-Seq data with ROADMAP DNAase1 data	https://www.encodeproject.org/data/annotations/v2/ Data file information: https://www.encodeproject.org/documents/a3631465-ac01-4a93-b257-88e344a4c3ef/@@download/attachment/ENCODEEncyclopediaV1README.pdf	2016/08/25
ROADMAP	All 127 ROADMAP epigenomes (15 chromatin states)	http://egg2.wustl.edu/roadmap/web_portal/chr_state_learning.html#core_15state	2016/08/25
Cattle 92 RNA-Seq datasets	79 different tissues from Dominette, the animal used for sequencing the reference bovine genome	http://www.ncbi.nlm.nih.gov/Traces/study/?acc=SRP049415	2016/08/25
FANTOM5 promoter atlas	All FANTOM phase 1 and phase 2 CAGE peaks	http://fantom.gsc.riken.jp/5/datafiles/latest/extra/CAGE_peaks/hg19.cage_peak_phase1and2combined_coord.bed.gz	2016/08/25
FANTOM5 enhancer atlas	All FANTOM5 permissive enhancers	http://enhancer.binf.ku.dk/presets/permissive_enhancers.bed	2016/08/25
SNP database	99.5 Million Cattle RefSNP	ftp://ftp.ncbi.nih.gov/snp/organisms/cow_9913/VCF/	Build 146 (Nov 24, 2015)
Villar datasets	MACS2 peak files and FASTQ files	https://www.ebi.ac.uk/arrayexpress/files/E-MTAB-2633/E-MTAB-2633.processed.1.zip (peak files)<br><br>https://www.ebi.ac.uk/arrayexpress/experiments/E-MTAB-2633/samples/ (FASTQ files)	2016/05/26
Vista enhancer datasets	1740 Vista validated human enhancers (hg19)	http://enhancer.lbl.gov/	2015/10/22
Cattle exon dataset	742,493 exons from UCSC exon annotation (hg19)	http://hgdownload.cse.ucsc.edu/goldenPath/hg19/database/knownGene.txt.gz	2016/06/27
liftOver chain files	Find relevant links for each species under the liftOver files	http://hgdownload.soe.ucsc.edu/downloads.html	

Supplementary Figures

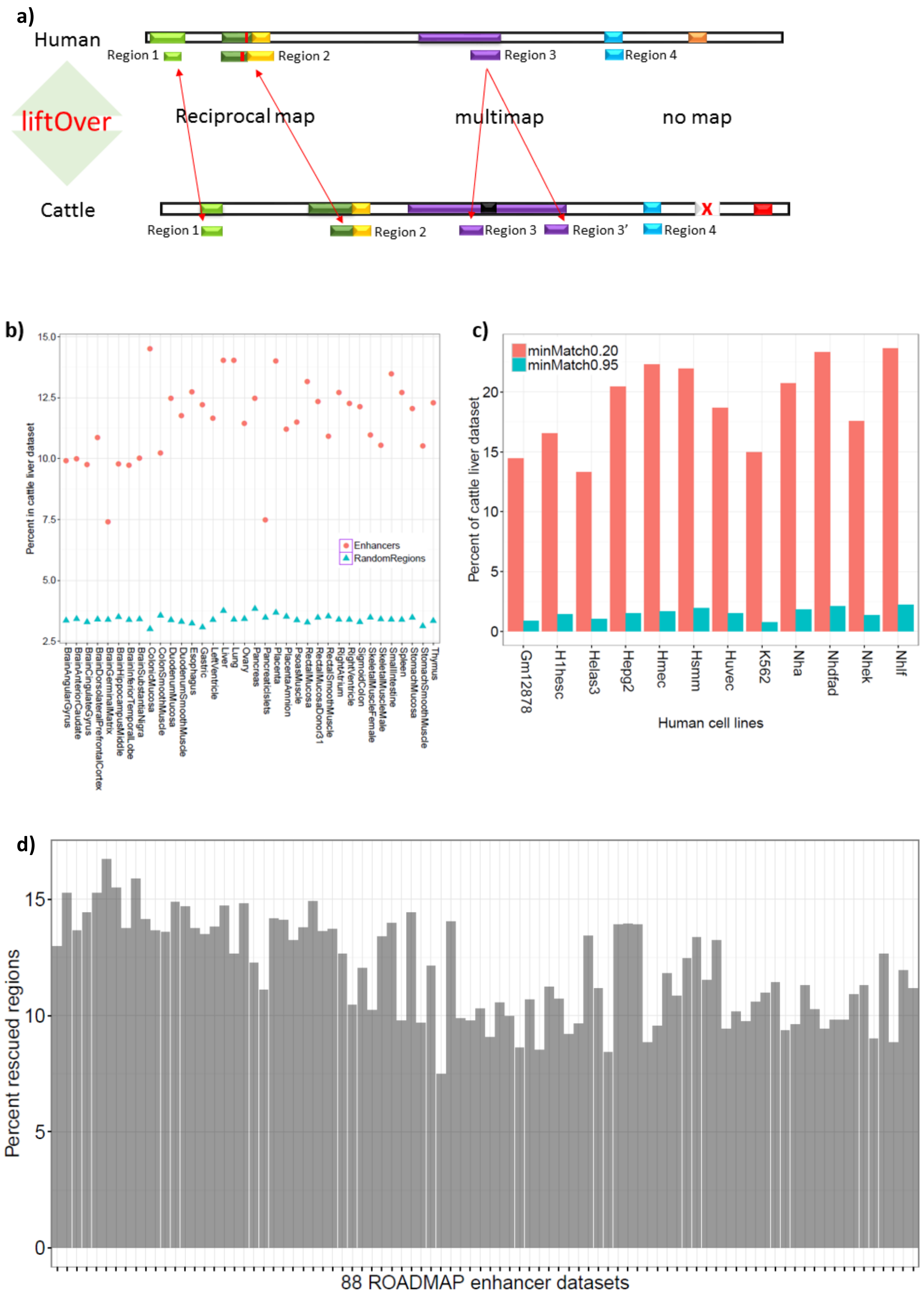

Fig. S1. Assessing mapping results for different human tissues and cell line datasets onto other species. The counts and percent are for mapped regions before the HPRS filtering pipeline. a) Possible cross-species mapping results, with three scenarios: (i) Reciprocal mapping with a low identity threshold (minMatch=0.20), but requires exact back mapping to human genome, (ii) Multiple mapping allowing multiple targets, but requiring a stringent minMatch=0.80, and (iii) Features that do not fall into the two categories above, such as species-specific enhancers, are not included in the prediction. b**)** Coverage of the cattle Villar enhancer reference dataset by predicted and random feature datasets. Features were mapped from 42 human enhancer datasets or 42 random datasets (equal number of regions and region length distribution) to the bovine genome and then compared for percent overlap with the cattle Villar reference liver enhancer dataset. For all 42 datasets, the regulatory datasets produced 5-10 times higher coverage of the reference bovine liver enhancers than the random datasets. c) Coverage of predictions by 12 common ENCODE human cell lines (x-axis). The number of recovered regions using minMatch = 0.2 and minMatch = 0.95 are shown. d) Percent of additional features that had multiple mapped targets, but met the high sequence similarity threshold for the reciprocal mapping from the bovine genome to the human genome (minMatch = 0.80).

a)

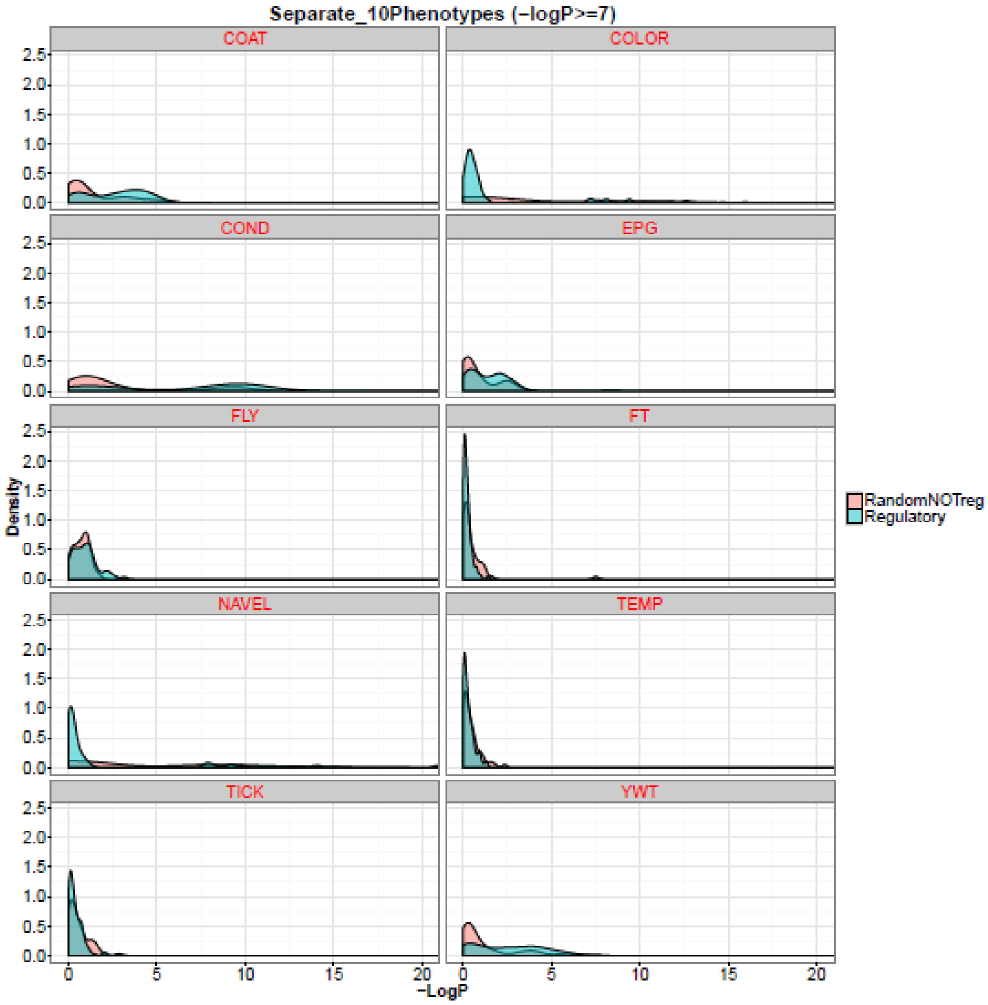
Separate_10Phenotypes (−logP>=7)
COAT
COLOR
COND
EPG
FLY
FT
NAVEL
TEMP
TICK
YWT
Density
−LogP
RandomNOTreg
Regulatory

b)

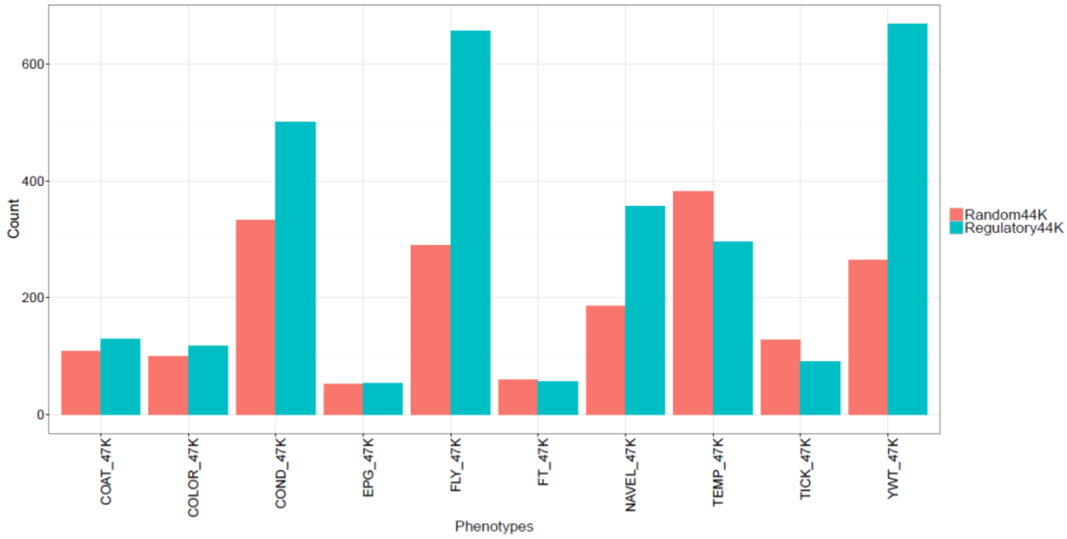
Count
Phenotypes
COAT_47K
COLOR_47K
COND_47K
EPG_47K
FLY_47K
FT_47K
NAVEL_47K
TEMP_47K
TICK_47K
YWT_47K
Random44K
Regulatory44K

Fig. S2. Enrichment of GWAS associated SNPs in HPRS filtered regulatory regions. Data are from a GWAS dataset for 2,112 cattle measured for ten different climatic change adaptation related phenotypes [6]. **a**) Number of GWAS significant SNPs, with –log(*P*-values) >= 7 in any of the 10 separate phenotypes. GWAS *P*-values for each trait <= 10^{-7} are considered significant with multiple test correction (with Bonferroni corrected P-value <= 0.05 and the number of SNP is ~500,000 SNPs). SNPs were selected for each of the 10 phenotypes separately and were pooled into one set and density plots of SNP counts and corresponding –log(*P*-values) for each phenotype are shown. X-axis shows –log(*P*-values) values from 0 to 20 and y-axis shows density of the SNP counts according to the –log*P* distribution. **b**) Significant SNPS were selected based on combined criteria: –log(*P*-values) > 2 and abs(effect size) >= the third quartile effect size value for each of the 10 phenotypes. The x-axis shows name IDs of the 10 phenotypes.

a)

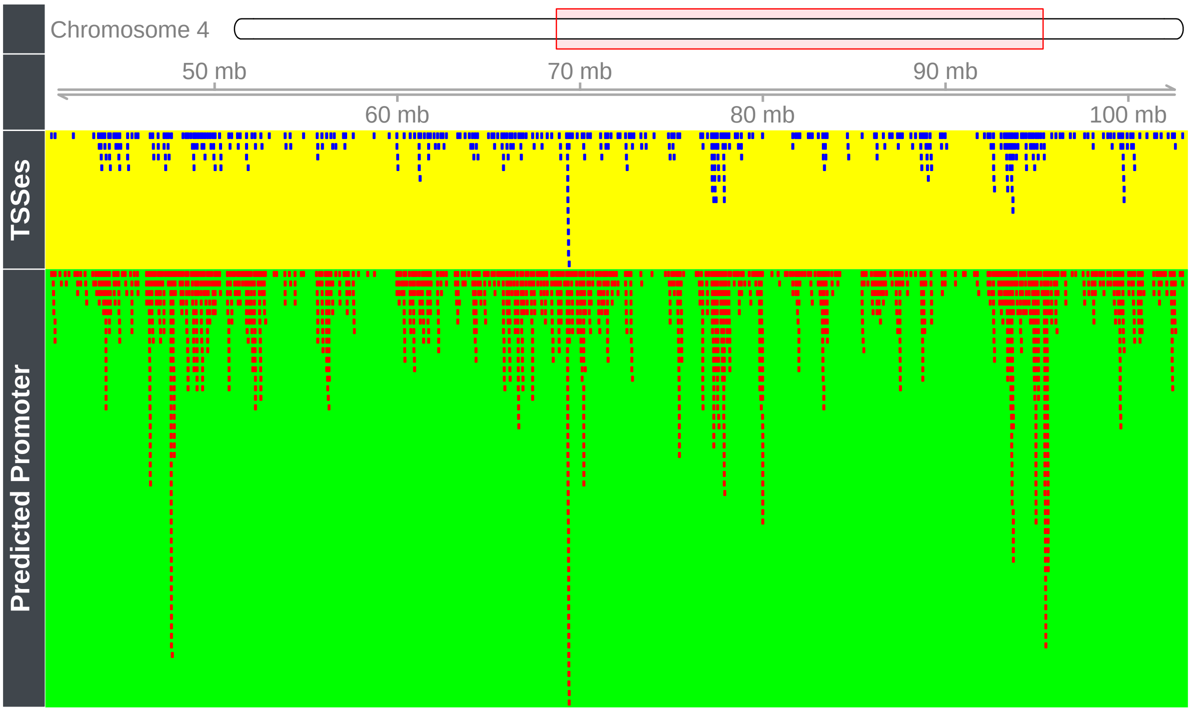
Chromosome 4
50 mb
70 mb
90 mb
60 mb
80 mb
100 mb
TSSes
Predicted Promoter

b)

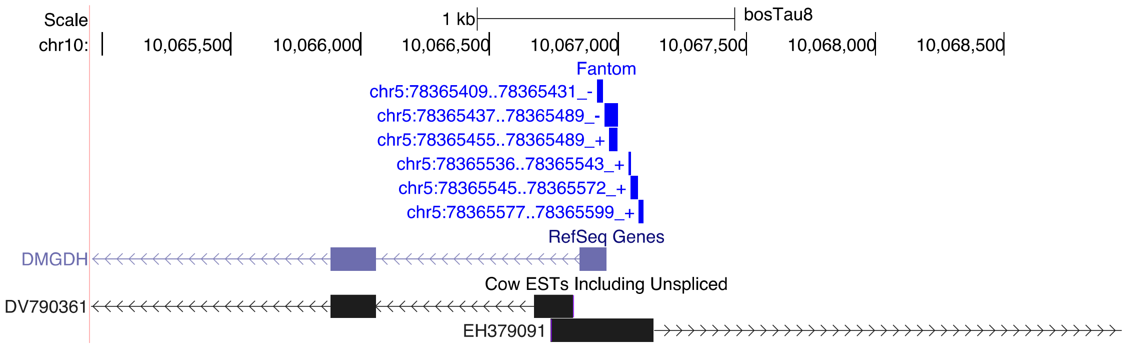
Scale
1 kb
bosTau8
chr10:
10,065,500
10,066,000
10,066,500
10,067,000
10,067,500
10,068,000
10,068,500
Fantom
chr5:78365409..78365431_-
chr5:78365437..78365489_-
chr5:78365455..78365489_+
chr5:78365536..78365543_+
chr5:78365545..78365572_+
chr5:78365577..78365599_+
RefSeq Genes
DMGDH
Cow ESTs Including Unspliced
DV790361
EH379091

c)

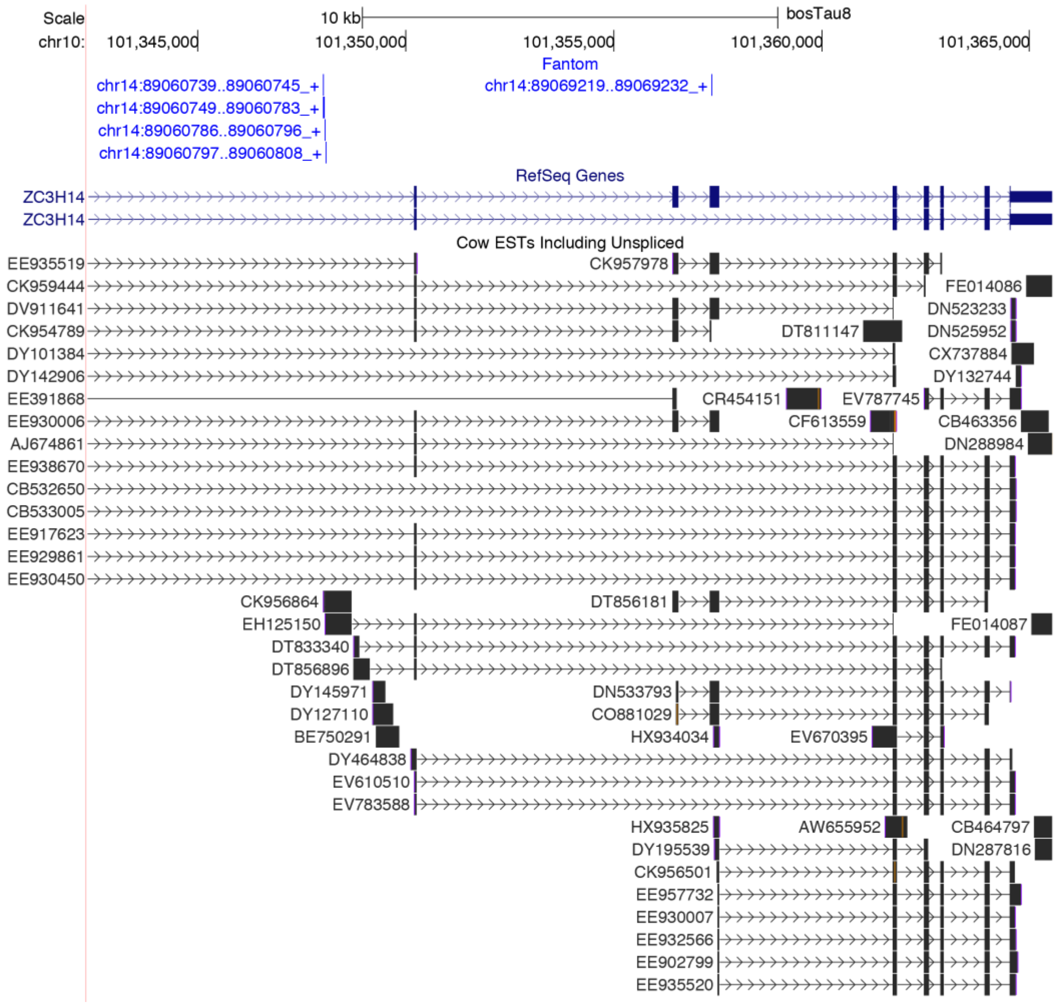

Fig. S3. Promoter prediction. a) We selected a random, large region of the chromosome to evaluate promoter prediction. We observed consistent overlapping of predicted promoters with known transcription start sites (TSSes). The higher and denser number of predicted promoters compared to annotated TSSes suggest that the HPRS prediction potentially led to the identification of unannotated promoters, including alternative promoters within annotated transcripts and promoters of unannotated transcripts such as those for long noncoding RNAs. b) HPRS promoters also predict bidirectional promoters with high accuracy (- for antisense, + for sense). c) HPRS predicted alternative promoters are supported by cattle expression sequencing tag (EST) data. The predicted promoters overlap the start sites of EST transcripts within the full length ZC3H14 gene.

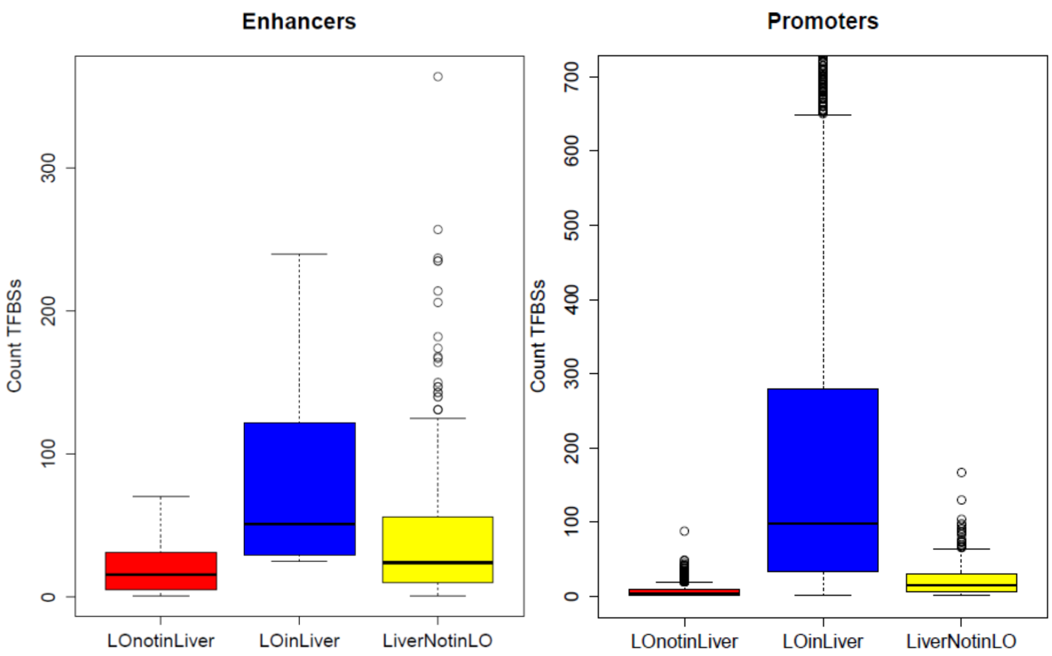

Fig. S4. Enrichment of TFBSs within enhancers and promoters. The promoters and enhancers were mapped from the human FANTOM enhancer [1] and the human FANTOM promoter databases onto the bovine genome [2]. Mapped regions were compared to the Villar bovine enhancer and promoter reference datasets for liver tissues [7]. Three categories of overlapping to the reference datasets were compared (x-axis): (i) mapped regions overlapping the Villar reference dataset (LOinLiver); (ii) mapped regions not in the Villar dataset (LOnotinLiver), and; (iii) regions in reference datasets not covered by mapped regions (LiverNotinLO). The TFBSs were derived from the whole bovine genome scanning using the Cluster Buster program [13] and three major transcription factor position weight matrix databases (TRANSFAC, JASPAR, and ENCODE) [21-23].

a)

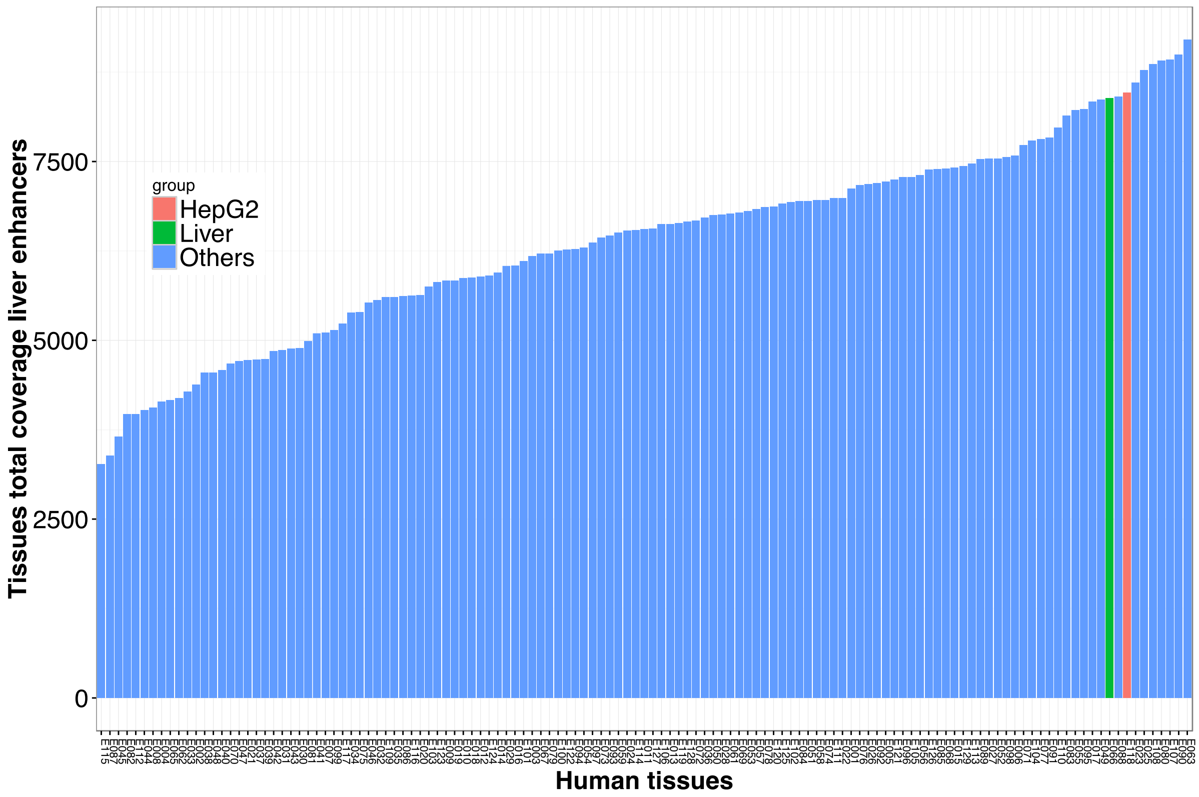
Tissues total coverage liver enhancers
7500
5000
2500
0
group
HepG2
Liver
Others
Human tissues

b)

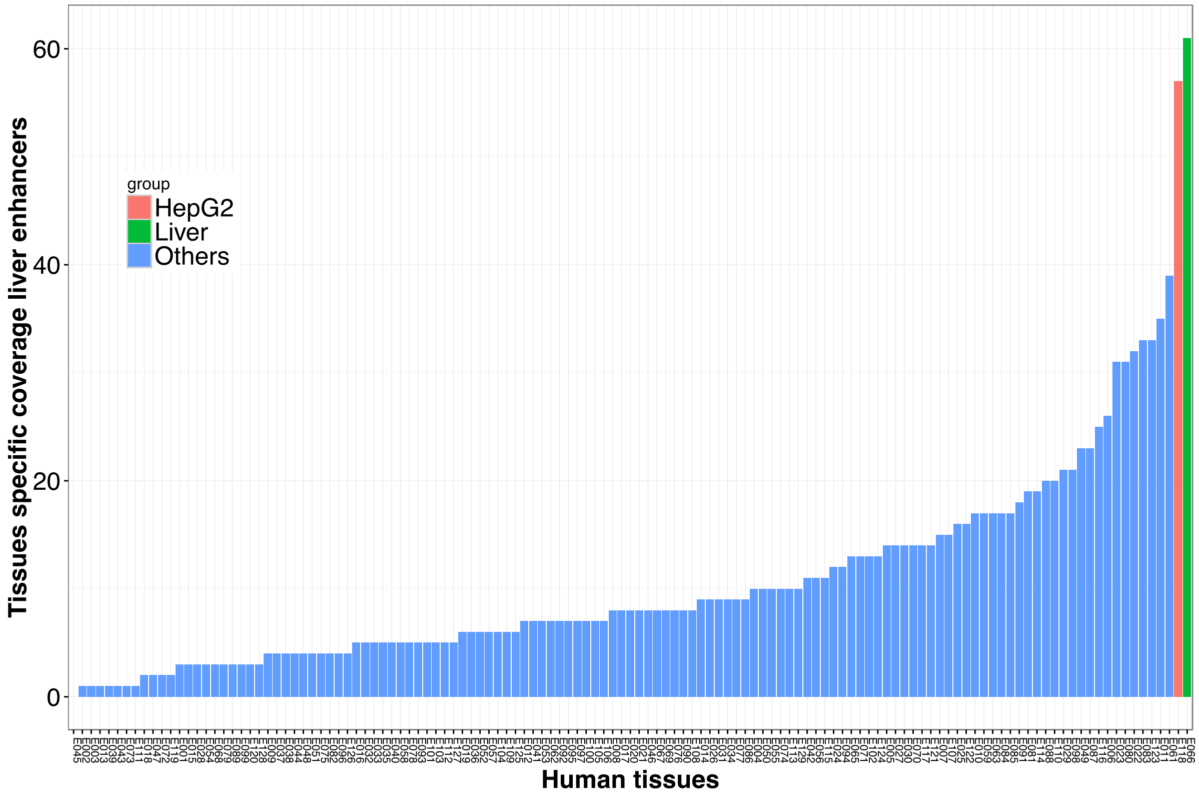
Tissues specific coverage liver enhancers
60
40
20
0
group
HepG2
Liver
Others
Human tissues

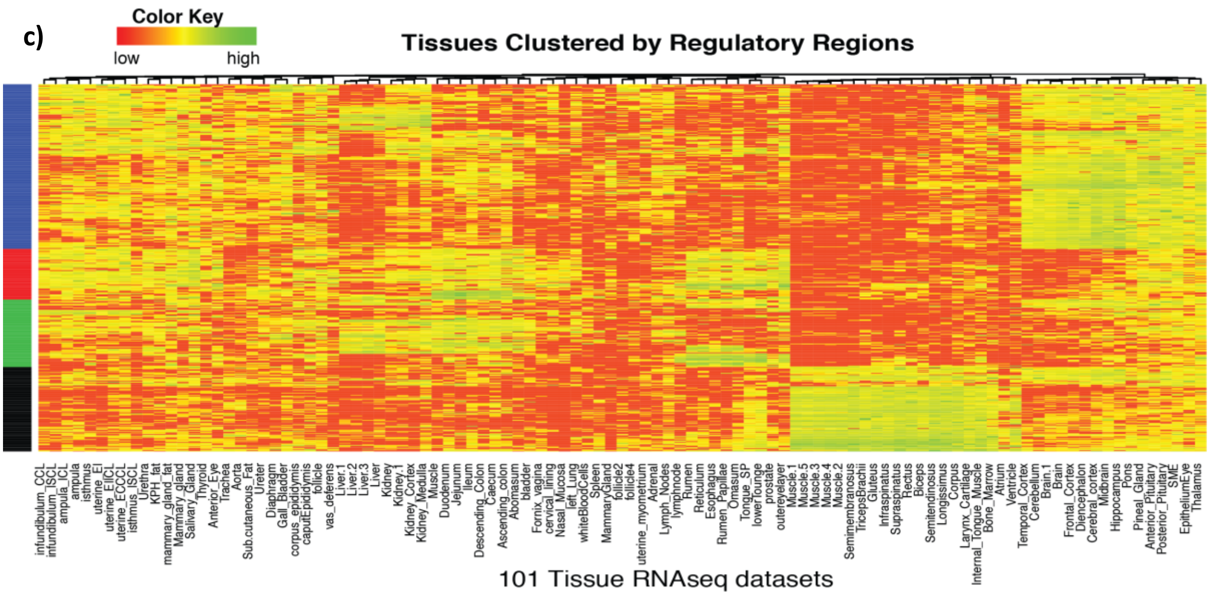

Fig. S5. Tissue specificity of predicted regulatory regions. a) Counts of HPRS predicted enhancers (using 88 ROADMAP human enhancer datasets) that overlap with the Villar cattle reference enhancers. b) We then defined tissue specific enhancer dataset by identifying HPRS regions that overlap with Villar reference enhancers for cattle and are unique for each of the 88 tissues. The datasets that yielded the highest overlap are those from the liver cell line (liver hepatocellular cells - HepG2) and the human liver tissue. c) We mapped 101 RNA sequencing datasets, collected from over 79 tissues (Table S5), to the predicted regulatory regions. The mapped RNA signal was used to compare the similarity between different tissues. Strong enrichment of brain, muscle, and liver tissues was observed.

a)

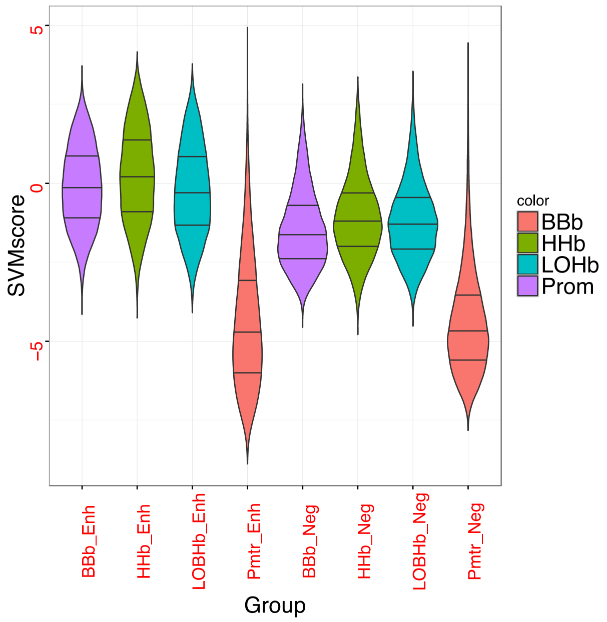

SVMscore
Group
5
0
-5
BBb_Enh
HHb_Enh
LOBHb_Enh
Pmtr_Enh
BBb_Neg
HHb_Neg
LOBHb_Neg
Pmtr_Neg
color
BBb
HHb
LOHb
Prom

b)

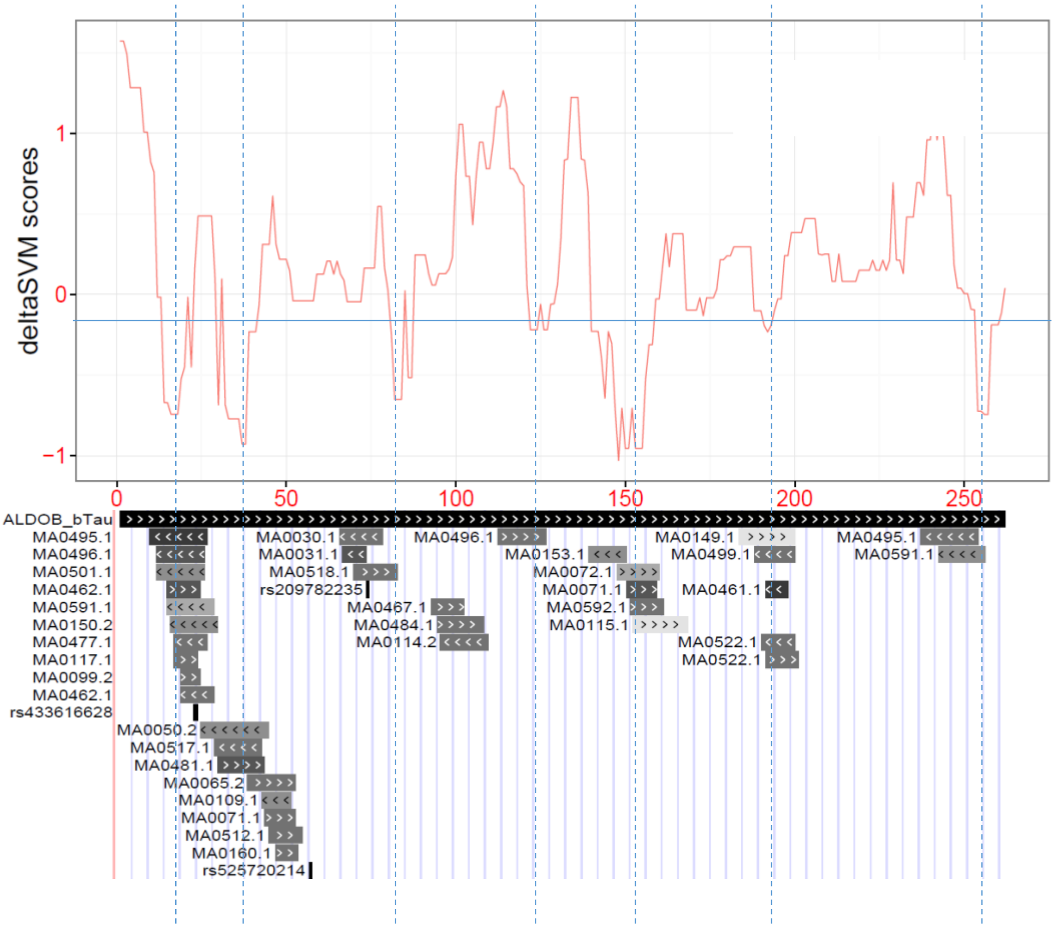

deltaSVM scores
1
0
−1
0
50
100
150
200
250
ALDOB_bTau
MA0495.1
MA0496.1
MA0501.1
MA0462.1
MA0591.1
MA0150.2
MA0477.1
MA0117.1
MA0099.2
MA0462.1
rs433616628
MA0050.2
MA0517.1
MA0481.1
MA0065.2
MA0109.1
MA0071.1
MA0512.1
MA0160.1
rs525720214
MA0030.1
MA0031.1
MA0518.1
rs209782235
MA0467.1
MA0484.1
MA0114.2
MA0496.1
MA0153.1
MA0072.1
MA0071.1
MA0592.1
MA0115.1
MA0149.1
MA0499.1
MA0461.1
MA0522.1
MA0522.1
MA0495.1
MA0591.1

Fig. S6. LS-gkm-SVM (large scale gapped k-mer support vector machine) scores for enhancers and deltaSVM scores for SNPs. a) The LS-gkm-SVM model was used to calculate the gkm-SVM scores for all enhancers in the Villar dataset. Red, enhancers scored on “enhancers versus background matrix”; green, random regions (selected by shuffling through the genomes to sample genomic regions of the same length to the Villar reference bovine enhancers) scored on “enhancers versus background matrix”; blue, enhancers scored on a “background versus background” matrix. The positive background was selected from the Villar reference enhancer dataset as described in the Supplementary Materials and Methods section. Training datasets using human (HHb) and cattle (BBb) and liftOver enhancer regions from human to cattle (LOBHb) yielded consistent and comparable results, which predicted higher scores for enhancer regions (BBb_Enh, HHb_Enh, LOBHb_Enh) than prediction for promoter (pmtr_Enh) and for random regions (BBb_Neg, HHb_Neg, LOBHb_Neg, and Pmtr_neg). **b)** deltaSVM for scoring SNP effects on enhancer activity. The LS-gkm-SVM model was used to score every possible SNP across the enhancer of the *ALDOB* gene (aldolase B fructose bisphosphate) in cattle. Single nucleotide resolution scores within the *ALDOB* enhancer are shown. Negative scores indicate loss of function (or TF binding), while positive scores indicate increases in activities. Computational predictions of transcription factor binding sites (by FIMO [29] and JASPAR position weight matrices) are shown in the lower panels. Transcription factor IDs and SNP IDs are shown next to the predicted regions. The *ALDOB* enhancer was mapped from humans to cattle. Vertical dashed lines show the locations of the deltaSVM peaks, where SNPs most likely reduce the enhancer activity, compared to the locations of predicted TFBSs. The deltaSVM score prediction was consistent with luciferase activity measurement (in humans) and to prediction of TFBSs (in humans and cattle).

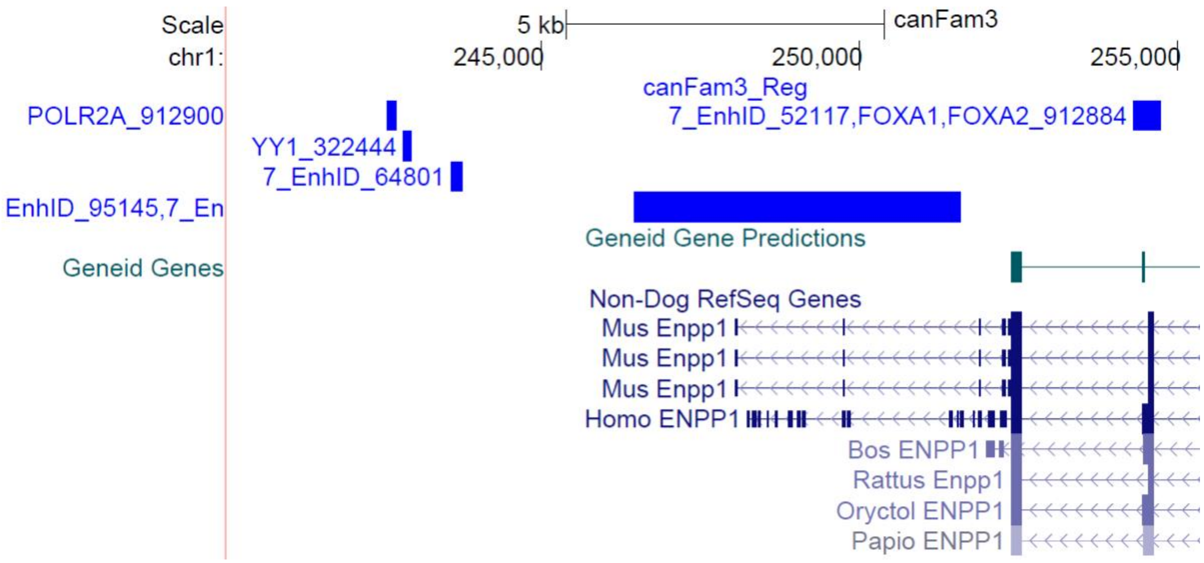

Fig. S7. An example of a simple view of the datasets generated for 10 mammalian species. The example is from the dog (canFam3) genome. Predicted regulatory regions are shown in blue with annotations (enhancer, promoter and transcription factor IDs) marked on the left. For regions with multiple annotations users can display the annotations by selecting the region on the browser. The example shows the *ENPP1* gene.

References

1. Bickhart, D.M. and G.E. Liu, *Identification of Candidate Transcription Factor Binding Sites in the Cattle Genome.* Genomics, Proteomics & Bioinformatics, 2013. **11**(3): p. 195-198.
2. Frith, M.C., M.C. Li, and Z. Weng, *Cluster-Buster: Finding dense clusters of motifs in DNA sequences.* Nucleic Acids Res, 2003. **31**(13): p. 3666-8.
3. Mathelier, A., et al., *JASPAR 2014: an extensively expanded and updated open-access database of transcription factor binding profiles.* Nucleic Acids Research, 2013.
4. Matys, V., et al., *TRANSFAC® and its module TRANSCompel®: transcriptional gene regulation in eukaryotes.* Nucleic Acids Research, 2006. **34**(suppl 1): p. D108-D110.
5. Wang, J., et al., *Sequence features and chromatin structure around the genomic regions bound by 119 human transcription factors.* Genome Res, 2012. **22**(9): p. 1798-812.
6. Porto-Neto, L.R., et al., *The Genetic Architecture of Climatic Adaptation of Tropical Cattle.* PLoS ONE, 2014. **9**(11): p. e113284.
7. Clop, A., et al., *A mutation creating a potential illegitimate microRNA target site in the myostatin gene affects muscularity in sheep.* Nat Genet, 2006. **38**(7): p. 813-818.
8. Bidwell, C.A., et al., *New insights into polar overdominance in callipyge sheep.* Anim Genet, 2014. **45 Suppl 1**: p. 51-61.
9. Cockett, N.E., et al., *Polar overdominance at the ovine callipyge locus.* Science, 1996. **273**(5272): p. 236-8.
10. Tellam, R., et al., *Genes Contributing to Genetic Variation of Muscling in Sheep.* Frontiers in Genetics, 2012. **3**(164).
11. Freking, B.A., et al., *Identification of the single base change causing the callipyge muscle hypertrophy phenotype, the only known example of polar overdominance in mammals.* Genome Res, 2002. **12**(10): p. 1496-506.
12. Andersson, R., et al., *An atlas of active enhancers across human cell types and tissues.* Nature, 2014. **507**(7493): p. 455-461.
13. The Fantom Consortium, Riken PMI, and CLST, *A promoter-level mammalian expression atlas.* Nature, 2014. **507**(7493): p. 462-470.
14. Zhu, Y., et al., *Predicting enhancer transcription and activity from chromatin modifications.* Nucleic Acids Research, 2013.
15. Lam, M.T., et al., *Enhancer RNAs and regulated transcriptional programs.* Trends in biochemical sciences, 2014. **39**(4): p. 170-182.
16. Creyghton, M.P., et al., *Histone H3K27ac separates active from poised enhancers and predicts developmental state.* Proceedings of the National Academy of Sciences, 2010. **107**(50): p. 21931-21936.
17. Elsik, C.G., et al., *Bovine Genome Database: new tools for gleaning function from the Bos taurus genome.* Nucleic Acids Research, 2015.
18. Villar, D., et al., *Enhancer Evolution across 20 Mammalian Species.* Cell, 2015. **160**(3): p. 554-566.
19. Li, H. and R. Durbin, *Fast and accurate short read alignment with Burrows-Wheeler transform.* Bioinformatics, 2009. **25**(14): p. 1754-60.
20. Liao, Y., G.K. Smyth, and W. Shi, *featureCounts: an efficient general purpose program for assigning sequence reads to genomic features.* Bioinformatics, 2014. **30**(7): p. 923-30.
21. Lee, D., *LS-GKM: a new gkm-SVM for large-scale datasets.* Bioinformatics, 2016.
22. Lee, D., et al., *A method to predict the impact of regulatory variants from DNA sequence.* Nat Genet, 2015. **47**(8): p. 955-961.
23. Siepel, A., et al., *Evolutionarily conserved elements in vertebrate, insect, worm, and yeast genomes.* Genome Research, 2005. **15**(8): p. 1034-1050.
24. Patwardhan, R.P., et al., *Massively parallel functional dissection of mammalian enhancers in vivo.* Nat Biotech, 2012. **30**(3): p. 265-270.

25. Quinlan, A.R. and I.M. Hall, *BEDTools: a flexible suite of utilities for comparing genomic features*. Bioinformatics, 2010. **26**(6): p. 841-2.
26. Li, H., et al., *The Sequence Alignment/Map format and SAMtools*. Bioinformatics, 2009. **25**(16): p. 2078-9.
27. Cheng, Y., et al., *Principles of regulatory information conservation between mouse and human.* Nature, 2014. **515**(7527): p. 371-375.
28. Roadmap Epigenomics, C., et al., *Integrative analysis of 111 reference human epigenomes.* Nature, 2015. **518**(7539): p. 317-330.
29. Grant, C.E., T.L. Bailey, and W.S. Noble, *FIMO: scanning for occurrences of a given motif.* Bioinformatics, 2011. **27**(7): p. 1017-8.
